# Belowground and aboveground herbivory differentially affect the transcriptome in roots and shoots of maize

**DOI:** 10.1002/pld3.426

**Published:** 2022-07-22

**Authors:** Wenfeng Ye, Carlos Bustos‐Segura, Thomas Degen, Matthias Erb, Ted C. J. Turlings

**Affiliations:** ^1^ Laboratory of Fundamental and Applied Research in Chemical Ecology, Institute of Biology University of Neuchâtel Neuchâtel Switzerland; ^2^ Institute of Plant Sciences University of Bern Bern Switzerland

**Keywords:** belowground and aboveground defense interactions, *Diabrotica virgifera*, phytohormones, *Spodoptera frugiperda*, transcriptome, *Zea mays*

## Abstract

Plants recognize and respond to feeding by herbivorous insects by upregulating their local and systemic defenses. While defense induction by aboveground herbivores has been well studied, far less is known about local and systemic defense responses against attacks by belowground herbivores. Here, we investigated and compared the responses of the maize transcriptome to belowground and aboveground mechanical damage and infestation by two well‐adapted herbivores: the soil‐dwelling western corn rootworm *Diabrotica virgifera virgifera* (Coleoptera: Chrysomelidae) and the leaf‐chewing fall armyworm *Spodoptera frugiperda* (Lepidoptera: Noctuidae). In responses to both herbivores, maize plants were found to alter local transcription of genes involved in phytohormone signaling, primary and secondary metabolism. Induction by real herbivore damage was considerably stronger and modified the expression of more genes than mechanical damage. Feeding by the corn rootworm had a strong impact on the shoot transcriptome, including the activation of genes involved in defense and development. By contrast, feeding by the fall armyworm induced only few transcriptional changes in the roots. In conclusion, feeding by a leaf chewer and a root feeder differentially affects the local and systemic defense of maize plants. Besides revealing clear differences in how maize plants respond to feeding by these specialized herbivores, this study reveals several novel genes that may play key roles in plant–insect interactions and thus sets the stage for in depth research into the mechanism that can be exploited for improved crop protection.

**Significance statement:**

Extensive transcriptomic analyses revealed a clear distinction between the gene expression profiles in maize plants upon shoot and root attack, locally as well as distantly from the attacked tissue. This provides detailed insights into the specificity of orchestrated plant defense responses, and the dataset offers a molecular resource for further genetic studies on maize resistance to herbivores and paves the way for novel strategies to enhance maize resistance to pests.

## 1 INTRODUCTION

Plants have evolved constitutive and inducible defense mechanisms to protect themselves from the constant attack by root and shoot herbivores (Erb, Glauser, et al., 2012; Johnson et al., 2016; Mithöfer & Boland, 2012). Inducible defenses start with the recognition of herbivore‐associated molecular patterns (HAMPs) and are followed by the activation of signaling networks. Previous studies have highlighted the roles of Ca^2+^ ion flux, mitogen‐activated protein kinase (MAPK) cascades, reactive oxygen species (ROS), and phytohormone signaling pathways including jasmonic acid (JA), salicylic acid (SA), abscisic acid (ABA), and ethylene (ET) on the expression regulation of defense‐related genes, which result in the production of defensive compounds (Broekgaarden et al., 2015; Erb & Reymond, 2019; Schuman & Baldwin, 2016; Wu & Baldwin, 2010). The production of defensive secondary metabolites or proteins in plants is referred to as direct defense (Erb & Reymond, 2019). In addition, plants can defend indirectly by emitting herbivore‐induced volatiles that attract natural enemies of the herbivores (Turlings & Erb, 2018) or producing resources for “bodyguards” such as extrafloral nectar (Heil, 2015). Well‐adapted herbivores may produce effectors that suppress plant defenses (Mutti et al., 2008; Ye et al., 2017) and even exploit plant defensive metabolites as foraging cues (Humphrey et al., 2016; Köhler et al., 2015; Machado et al., 2021; Miles et al., 2005; Renwick & Lopez, 1999) and/or sequester them for their own protection (Kos et al., 2011; Kumar et al., 2014; Robert et al., 2012; Singer et al., 2009; Smilanich et al., 2009; Sternberg et al., 2012).

Shoot herbivory induces defenses in both leaves and roots. For example, larval performance of western corn rootworm *Diabrotica virgifera virgifera* is attenuated by previous leaf herbivory by fall armyworm *Spodoptera frugiperda* caterpillars (Erb, Robert, et al., 2011). Similarly, leaf attack by diamondback moth caterpillars *Plutella xylostella* strongly reduces the performance of cabbage root fly larvae *Delia radicum* feeding on roots of cabbage plants *Brassica oleracea* (Karssemeijer et al., 2020). In maize, aboveground herbivory by cotton leafworm *Spodoptera littoralis* does not induce JA in roots (Erb, Flors, et al., 2009). By contrast, an increase in jasmonate levels has been observed in roots of tobacco plants 2 h after leaves were mechanically damaged and oral secretion (OS) from tobacco hornworm *Manduca sexta* was added to the wounds (Machado et al., 2018). Cabbage plants also increase JA in roots in response to aboveground herbivory by caterpillars, but not by aphids (Karssemeijer et al., 2020). These plant‐mediated interactions can lead to defense facilitation but also suppression, like in tallow trees, where different aboveground herbivores induce diverse defensive responses, including the differential synthesis of metabolites in roots (Huang et al., 2014; Xiao et al., 2019). Aboveground adults of the tallow tree specialist beetle *Bikasha collaris* thus facilitate development of conspecific belowground larvae, but heterospecific aboveground herbivory may inhibit *B. collaris* larval development (Huang et al., 2014). Thus, the induction of root defenses by shoot herbivory can be highly plant‐ and herbivore‐specific.

Compared with the well‐studied inducible defense mechanism aboveground, less is known about the belowground defense of plants against root herbivores (Erb, Glauser, et al., 2012). As in shoots, the responses of plant roots to herbivore attack are insect‐specific (Rasmann & Turlings, 2008) and different from artificial damage (Lu et al., 2015). JA is the most important phytohormone that mediates plant defense against chewing herbivores (Erb, Meldau, et al., 2012; Howe & Jander, 2008; Wu & Baldwin, 2010) and is involved in the activation of both local and systemic defenses (Bozorov et al., 2017; Lortzing & Steppuhn, 2016). However, the regulation of the JA pathway differs significantly between roots and shoot (Acosta et al., 2013). Belowground and aboveground herbivore attack induces the jasmonate production both in roots (Erb, Flors, et al., 2009; Lu et al., 2015) and shoots (Erb, Flors, et al., 2009; Erb, Meldau, et al., 2012; Wu & Baldwin, 2010), but jasmonates are less inducible in the roots than in the leaves in response to herbivory and mechanical wounding (Erb, Flors, et al., 2009; Hasegawa et al., 2011; Tretner et al., 2008). In contrast to herbivore‐attacked leaves, there is, at least so far, no strong evidence for a role of SA, ABA and ethylene in defenses against root herbivory (Erb, Flors, et al., 2009; Johnson et al., 2016; Lu et al., 2015), and nothing is known about the involvement of other phytohormones in root defense mechanisms. A notable recent study shows that root herbivory by *D. radicum* changes the expression of ABA and ethylene biosynthesis genes in cabbage roots after 24 h, suggesting the potential role of these phytohormones in later stages of the defense response (Karssemeijer et al., 2020). Root herbivory not only induces reconfiguration of primary metabolites in roots (Lu et al., 2015; Pan et al., 2020), but it also activates systemic physiological changes aboveground. For instance, plants infested with root herbivores reallocated carbon (Robert et al., 2014) and nitrogen (Tao & Hunter, 2013) to the shoots. Belowground herbivory by *D. v. virgifera* induces water stress, resulting in the accumulation of ABA in maize shoots, and enhanced resistance against chewing leaf herbivores (Erb, Köllner, et al., 2011). Over all, there are still large gaps in our understanding of the mechanism of root‐herbivory‐induced shoot defense.

In response to herbivore attack, maize plants accumulate defense proteins and toxic secondary metabolites. For example, the transcription level of defense‐related genes coding for maize proteinase inhibitor (MPI), cystatin‐like proteinase inhibitor, and serine protease inhibitor is induced by *S. littoralis* infestation (Ton et al., 2007). MPI inhibits the activity of digestive enzymes in the gut of *S. littoralis* (Tamayo et al., 2000). Benzoxazinoids (BXs), a major group of indole‐derived secondary metabolites, have a well‐established role in defense against herbivory in maize (Frey et al., 2009). BX biosynthesis pathway and enzymes that function in the BX production are comprehensively documented (Frey et al., 2009; Tzin et al., 2017). In maize leaves, the content of BXs and transcript levels of BX biosynthetic genes are highly induced locally in response to caterpillar feeding. BXs such as DIMBOA and HDMBOA are toxic and repellent to *S. littoralis*, respectively (Glauser et al., 2011). Moreover, *Spodoptera exigua* and *S. littoralis* caterpillars perform considerably better on maize BX‐deficient mutants (Maag et al., 2016; Tzin et al., 2017). The larger amounts of BXs in maize crown roots compared with primary roots play a role in deterring feeding by generalist herbivores (Robert et al., 2012). In contrary, well‐adapted herbivores such as *D. v. virgifera* and *S. frugiperda* have been shown to tolerate high concentrations of benzoxazinoids and use them as foraging cues (Köhler et al., 2015; Robert et al., 2012).

In addition to non‐volatile defense metabolites, maize plants also emit blends of volatile organic compounds (VOCs) that can act as repellents of the herbivores (Bernklau et al., 2016), foraging cues to natural enemies of the pests (Dicke & Sabelis, 1988; Rasmann et al., 2005; Tamiru et al., 2011; Turlings et al., 1990), or airborne signals in systemic defense and plant–plant communication (Engelberth et al., 2004; Erb et al., 2015; Ton et al., 2007). Volatile indole, for instance, has been shown to prime defenses in maize plants (Erb et al., 2015). As for direct defense responses, the molecular mechanisms that are involved in this multifunctional volatile signaling remain to be elucidated.

While considerable information about aboveground and belowground defense responses to herbivory is available, few studies so far have directly compared transcriptional responses of roots and shoots in response to damage and herbivore attack. To fill this knowledge gap, we characterized the local and systemic transcriptional changes of maize responses to belowground infestation by *D. v. virgifera* larvae and aboveground herbivory by *S. frugiperda* caterpillars and compared them with mechanical damage. The resulting dataset provides extensive insights into the specificity and orchestration of root and shoot defense responses to herbivore attack.

## 2 RESULTS

### 2.1 Overview of transcriptional changes in maize plants in response to belowground and aboveground insect herbivory

To investigate the global transcriptomic changes that occur in response to aboveground and belowground insect herbivory, maize plants (var. Delprim) were either infested for 72 h by root feeding *D. v. virgifera* larvae, leaf feeding *S. frugiperda* larvae, or damaged mechanically on roots or shoots. The expression levels of eight selected genes were confirmed by qRT‐PCR to validate the RNA‐seq results. Similar expression patterns and high correlation coefficients of qRT‐PCR and FPKM data (Figure S1) confirmed the reliability of the RNA‐seq data. Detailed information on RNA sequencing and mapping is provided in Table S1.

**FIGURE 1:**
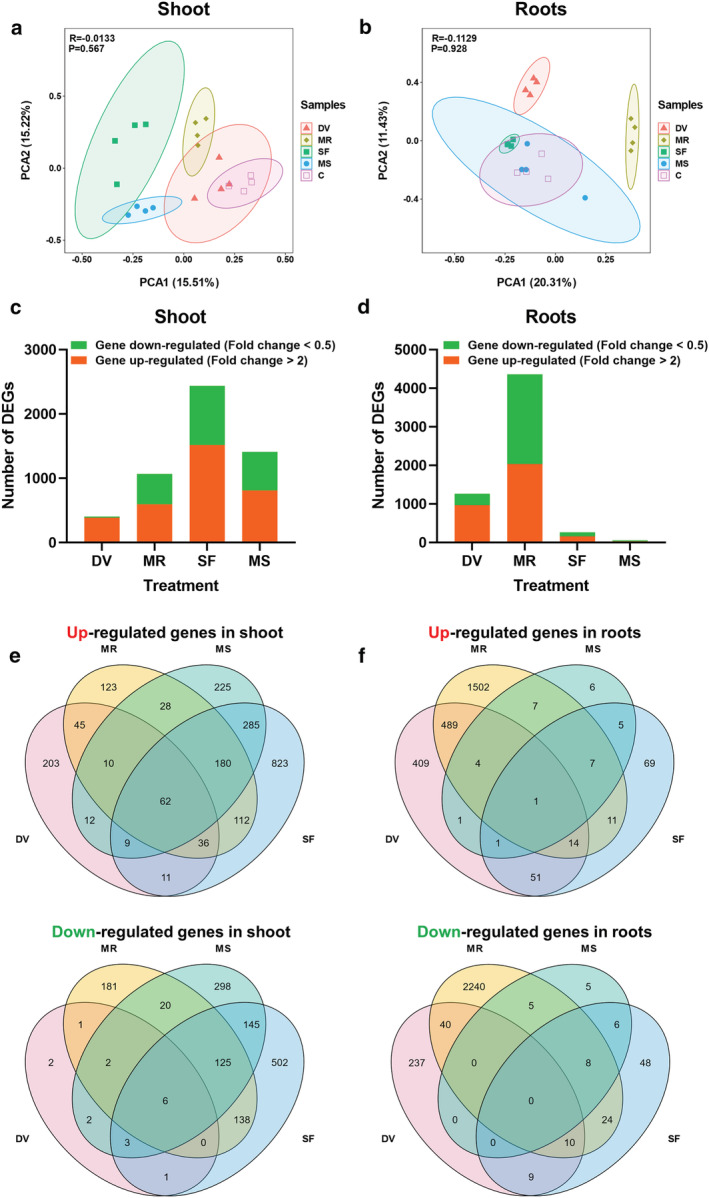
Overview of maize transcriptome responses to belowground and aboveground insect herbivory. (a and b) PCA plots of transcripts identified by RNA‐seq of maize shoot (a) and roots (b) from seedlings after 72 h of belowground infestation by *Diabrotica virgifera virgifera* (DV) or aboveground infestation by *Spodoptera frugiperda* (SF), or after application of mechanical root (MR) or shoot damage (MS). Non‐treated seedlings served as controls (C). (c and d) Total number of transcripts that were significantly upregulated or downregulated in maize shoot (c) and roots (d) after each treatment compared with non‐manipulated controls. (e and f) Venn diagrams illustrating the number of transcripts upregulated or downregulated in shoot (e) and roots (f) in response to belowground and aboveground treatments

Of 46,430 predicted genes in the B73 V4 reference genome, a total of 37,997 detectable corresponding transcripts could be identified across all samples (Data S1). Principal component analyses (PCA) revealed that in the shoots, the gene expression profiles of control plants were clearly separated from *S. frugiperda*‐infested, leaf wounded and root wounded plants, but overlapping with those of *D. v. virgifera*‐infested plants (Figure 1a). Principal component analyses (PCA) of the root data show that the gene expression profiles in control root samples were separated from *D. v. virgifera*‐infested and root wounded plants, but not from shoot wounded and *S. frugiperda*‐infested plants (Figure 1b). Thus, it appears that local responses are generally more pronounced than systemic responses, and herbivory elicits specific regulation patterns relative to mechanical wounding.

Genes with a false discovery rate (FDR) adjusted *P* < .05 and an absolute value of log_2_‐transformed fold change (treatment/control) > 1 were selected as differentially expressed genes (DEGs) for further analysis. Shoot samples from *D. v. virgifera*‐infested, root‐artificially damaged, *S. frugiperda*‐infested and leaf‐artificially damaged plants exhibited 405 (388 up and 17 down), 1069 (596 up and 473 down), 2438 (1518 up and 920 down), and 1412 (811 up and 601 down) DEGs, respectively (Figure 1c, Data S2, S3, S4, and S5). Root samples from *D. v. virgifera*‐infested and root‐mechanically wounded plants exhibited 1266 (970 up and 296 down) and 4362 (2035 up and 2327 down) DEGs, respectively, whereas *S. frugiperda*‐infestation and leaf‐mechanical wounding induced only 264 (159 up and 105 down) and 56 (32 up and 24 down) DEGs in root samples, respectively (Figure 1d, Data S2, S3, S4 and S5). Compared with control plants, both belowground and aboveground insect herbivory induced local transcriptional changes, with systemic changes being less pronounced (Figure 1c,d). Local mechanical damage also elicited local responses and weaker systemic responses. Interestingly, leaf herbivore attack triggered stronger responses than mechanical shoot damage, while the opposite was the case for root herbivore attack and mechanical root damage, where the damage treatment led to stronger responses (Figure 1c,d). The distribution of upregulated and downregulated DEGs in maize shoots and roots in response to each treatment was calculated and presented in Venn diagrams (Figure 1e,f). In maize leaves, the expression of a small number of genes was regulated (62 up and 6 down) by all treatments. Two sets of genes were specifically regulated by aboveground *S. frugiperda* herbivory (823 up and 502 down) and belowground *D. v. virgifera* infestation (203 up and 2 down) (Figure 1e). In the roots, a total of 117 genes (69 up and 48 down) were specifically regulated by *S. frugiperda* herbivory, and 646 genes (409 up and 237 down) were specifically regulated by *D. v. virgifera* infestation (Figure 1f). Thus, both local and systemic responses are highly specific.

### 2.2 Differential expression of genes in plants attacked by *S. frugiperda*

The DEGs of maize transcriptome in response to aboveground and belowground herbivory were further subjected to KEGG pathway enrichment analysis to identify pathways that are differentially regulated. The DEGs in maize shoots that responded to *S. frugiperda* attack were assigned to 42 significant KEGG pathways (adjusted *P* < .05) (Data S6), of which the top 20 enriched pathways are presented in Figure S2A (global and overview maps pathways were excluded). The biosynthesis of phenylpropanoids, flavonoids, and benzoxazinoids and the metabolism of α‐linolenic acid, as well as other metabolic pathways associated with plant defense, signal transduction, and primary metabolism, showed strong changes in maize shoots after *S. frugiperda* herbivory (Figure S2A and Data S6). When comparing *S. frugiperda* herbivory and artificial shoot damage, the DEGs are mainly involved in energy metabolism, such as the biosynthesis of carbohydrates, lipids, and amino acids. Several DEGs are also involved in the biosynthesis of certain secondary metabolites and the transduction of plant hormone signals (Figure S2B and Data S6). DEGs in maize roots that responded to *S. frugiperda* attack were assigned to 11 significant pathways, mainly involving the biosynthesis of phenylpropanoid and flavonoids, and some primary metabolism pathways including the metabolism of amino acids, nitrogen, and carbohydrates (Figure S2C and Data S6). Notably, shoot and root responses to *S. frugiperda* attack comprised the biosynthesis of phenylpropanoids, flavonoids, and benzoxazinoids as well as phenylalanine, tyrosine, and tryptophan (Figure S2A,C and Data S6), implying the potential role of these pathways in general systemic stress responses to herbivory. Figure S3 and Data S7 provide detailed information on the 60 most upregulated genes and 60 most downregulated genes in the shoot and in response to *S. frugiperda* herbivory.

### 2.3 Differential expression of genes in plants attacked by *D. v. virgifera*

The DEGs in maize roots that responded to *D. v. virgifera* attack were assigned to 52 significant KEGG pathways (adjusted *P* < .05) (Data S6), and the top 20 enriched pathways are presented in Figure S4A (global and overview maps pathways were excluded). *D. v. virgifera* herbivory strongly induced the pathways involved in the metabolism of phenylpropanoid, α‐linolenic acid, and monoterpenoids, as well as primary pathways involved in the metabolism of amino acids, lipids, and carbohydrates (Figure S4A and Data S6). Most DEGs associated with the biosynthesis of jasmonic acid and methyl jasmonate in the α‐linolenic acid metabolism pathway were upregulated in response to *D. v. virgifera* infestation. Of the plant hormone signal transduction pathways, genes associated with JA signaling transduction (*JASMONATE ZIM*‐domain [*JAZ*] and *MYC2*) and genes responsible for disease resistance via SA signaling (transcription factor *TGA* and pathogenesis‐related protein 1 gene *PR1*) were upregulated by *D. v. virgifera* infestation (Data S2 and Data S6). These results suggest that both JA and SA signaling are involved in the defense responses of maize roots to *D. v. virgifera*. When comparing *D. v. virgifera* herbivory and artificial root damage, the DEGs are mainly those involved in phenylpropanoid biosynthesis, plant hormone signal transduction, plant–pathogen interaction, genetic information processing, and cellular processes. Several DEGs are also linked to primary metabolism pathways such as the metabolism of carbohydrates, amino acids, and lipids (Figure S4B and Data S6). DEGs in maize shoot, when comparing *D. v. virgifera* herbivory and the control treatment, were assigned to 13 relevant pathways involved in DNA replication, linoleic acid metabolism, translation, carotenoid biosynthesis, and other metabolisms of energy, carbohydrate, nucleotide, and amino acids (Figure S4C and Data S6). All DEGs involved in DNA replication were upregulated in shoot tissue in response to belowground *D. v. virgifera* herbivory, whereas DEGs in translation and carbon fixation were downregulated (Data S2 and Data S6). Figures S5 and Data S8 provide detailed information on the 60 most upregulated genes and 60 most downregulated genes in the root in response to *D. v. virgifera* feeding.

### 2.4 Plant hormone‐related genes induced by belowground and aboveground insect herbivory

**FIGURE 2:**
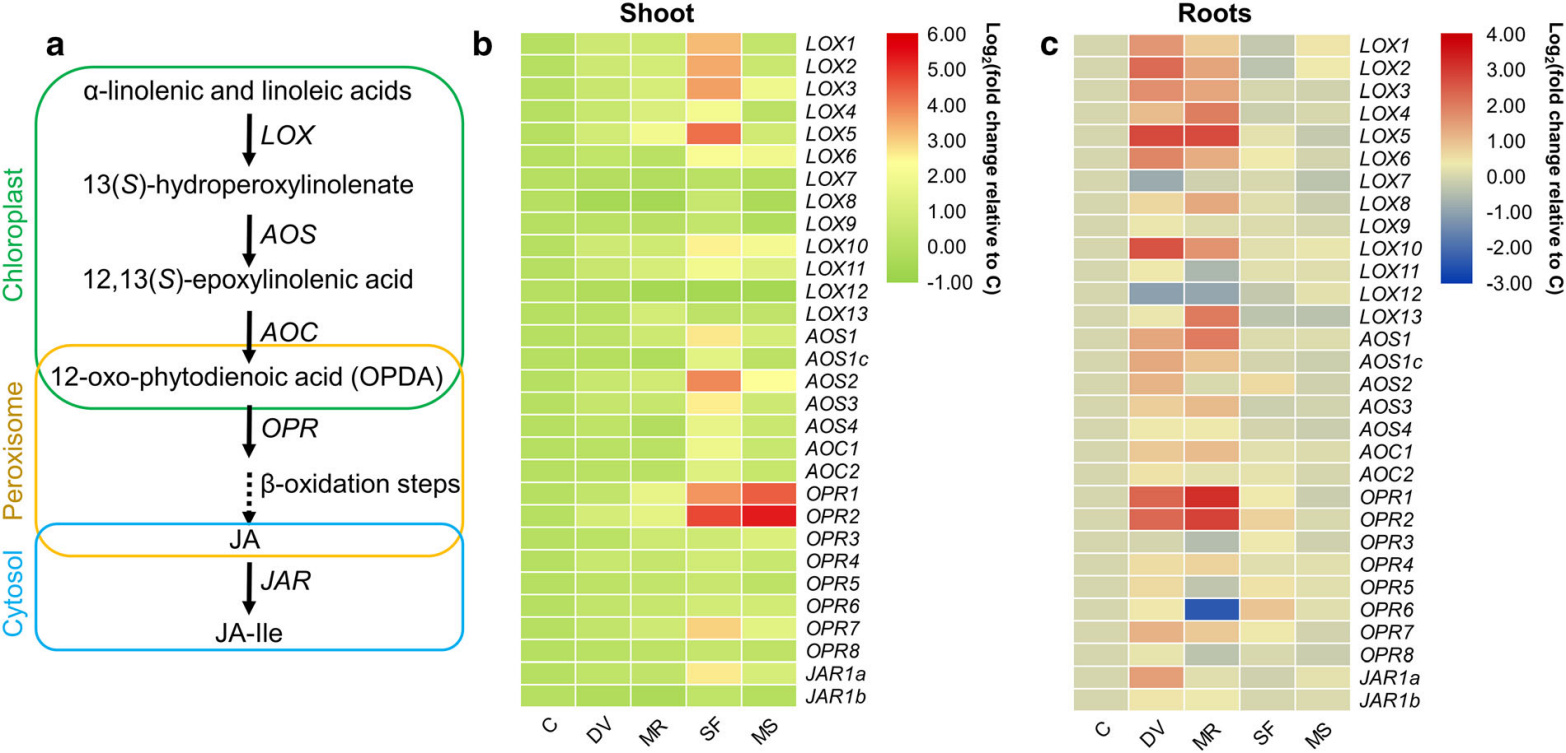
Effects of belowground and aboveground insect herbivory on jasmonic acid (JA) pathway gene expression. (a) Schematic diagram of the JA biosynthesis pathway. *LOX*, lipoxygenase; *AOS*, allene oxide synthase; *AOC*, allene oxide cyclase; *OPR*, 12‐oxophytodienoate reductase; *JAR*, jasmonate resistant; JA‐Ile, jasmonoyl‐isoleucine. The dashed arrow represents multiple enzymatic steps (Tzin et al., 2015). (b and c) Heat map of JA biosynthesis‐related gene expression in maize shoot (b) and roots (c). Samples were collected from maize plants that were kept non‐manipulated (C, control) or after 72 h of belowground infestation by *Diabrotica virgifera virgifera* (DV), mechanical damage on root (MR), 72 h of aboveground infestation by *Spodoptera frugiperda* (SF), or mechanical damage on shoot (MS). Color coding represents the range of log_2_(fold change relative to control).

**FIGURE 3:**
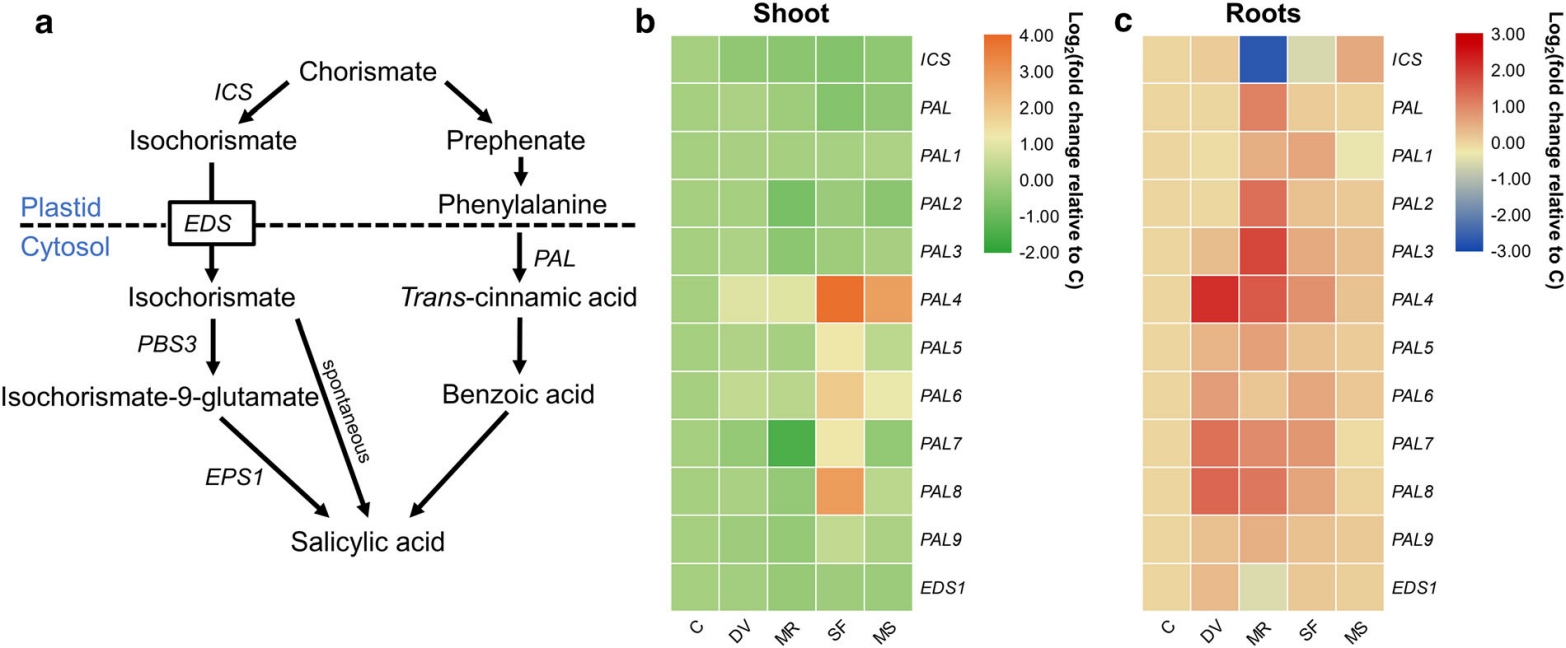
Effects of belowground and aboveground insect herbivory on salicylic acid (SA) pathway gene expression. (a) Schematic diagram of the SA biosynthesis pathway. *ICS*, isochorismate synthase; *PAL*, phenylalanine ammonia lyase; *EDS*, enhanced disease susceptibility; isochorismate is transported by the multidrug and toxin extrusion (MATE) transporter EDS to the cytosol. *PBS3*, avrPphB susceptible 3; *EPS1*, enhanced pseudomonas susceptibility 1. (b and c) Heat map of SA biosynthesis‐related gene expression in maize shoot (b) and roots (c). Samples were collected from maize plants that were kept non‐manipulated (C, control) or after 72 h of belowground infestation by *Diabrotica virgifera virgifera* (DV), mechanical damage on root (MR), 72 h of aboveground infestation by *Spodoptera frugiperda* (SF), or mechanical damage on shoot (MS). Color coding represents the range of log_2_(fold change relative to control).

**FIGURE 4:**
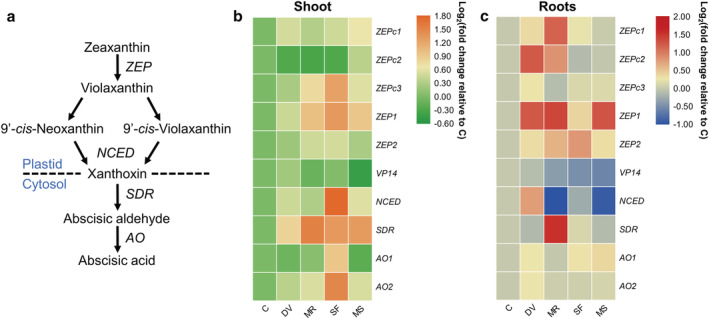
Effects of belowground and aboveground insect herbivory on abscisic acid (ABA) pathway gene expression. (a) Schematic diagram of the ABA biosynthesis pathway. *ZEP*, zeaxanthin epoxidase; *NCED*, 9‐*cis*‐epoxycarotenoid dioxygenase; *SDR*, short‐chain dehydrogenase/reductase; *AO*, aldehydeoxidase (Leng et al., 2014). (b and c) Heat map of ABA biosynthesis‐related gene expression in maize shoot (b) and roots (c). *VP14*, viviparous14, 9‐*cis*‐epoxycarotenoid dioxygenase 1. Samples were collected from maize plants that were kept non‐manipulated (C, control) or after 72 h of belowground infestation by *Diabrotica virgifera virgifera* (DV), mechanical damage on root (MR), 72 h of aboveground infestation by *Spodoptera frugiperda* (SF), or mechanical damage on shoot (MS). Color coding represents the range of log_2_(fold change relative to control).

**FIGURE 5:**
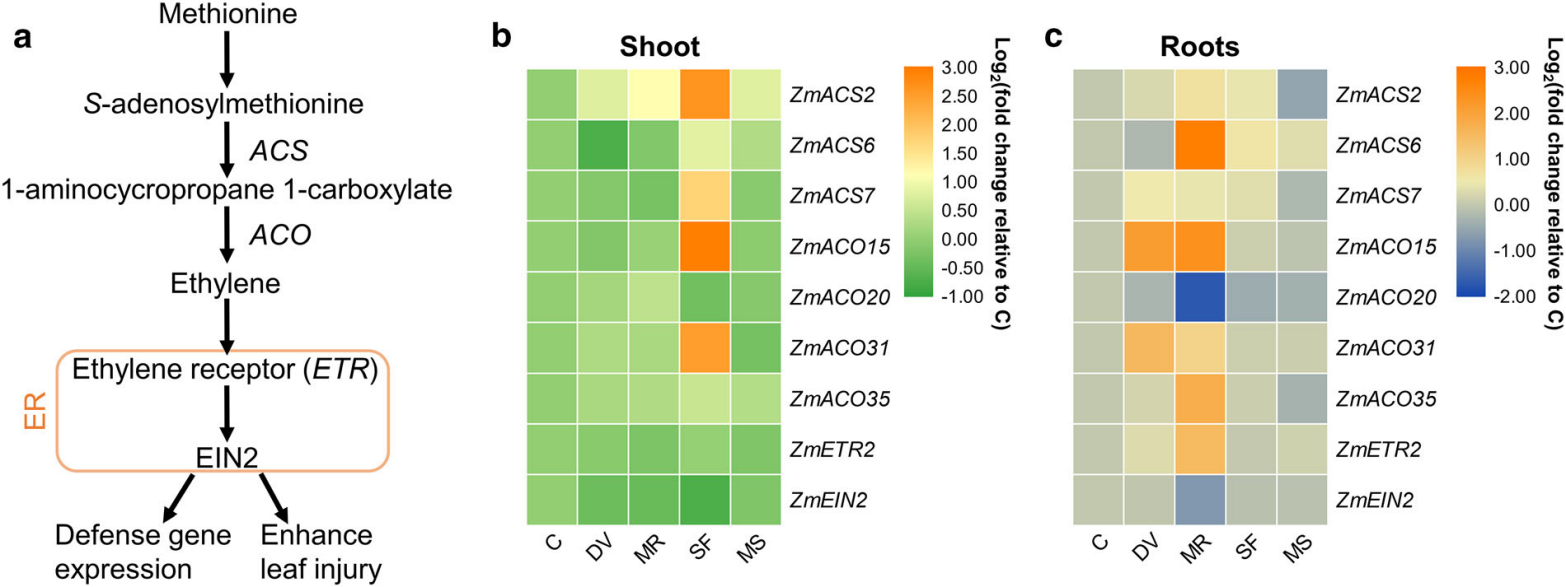
Effects of belowground and aboveground insect herbivory on ethylene pathway gene expression. (a) Schematic diagram of the ethylene signaling pathway. ER, endoplasmic reticulum; *ACS*, 1‐aminocyclopropane‐1‐carboxylate synthase; *ACO*, 1‐aminocyclopropane‐1‐carboxylate oxidase; *ETR*, ethylene receptor; *EIN2*, ethylene insensitive 2 (Tamaoki, 2008). (b and c) Heat map of ethylene signaling pathway‐related gene expression in maize shoot (b) and roots (c). Samples were collected from maize plants that were kept non‐manipulated (C, control) or after 72 h of belowground infestation by *Diabrotica virgifera virgifera* (DV), mechanical damage on root (MR), 72 h of aboveground infestation by *Spodoptera frugiperda* (SF), or mechanical damage on shoot (MS). Color coding represents the range of log_2_(fold change relative to control).

To determine phytohormone‐related gene expression changes in response to belowground and aboveground insect infestation, we compared the expression of genes associated with JA, SA, ABA, and ethylene biosynthesis in maize shoot and roots for the five plant treatments (Data S9). In general, the expression pattern of genes involved in JA (Figure 2), SA (Figure 3), ABA (Figure 4), and ethylene pathway (Figure 5) were highly induced locally in response to belowground and aboveground infestation or artificial damage, whereas root and shoot damage by insect herbivory and mechanical wounding also systemically induced the strong expression of ABA‐related genes (Figure 4).

Infestation of maize shoots by *S. frugiperda* induced the expression of JA‐related genes in shoot tissue to a greater extent than artificial leaf damage, especially in the first and second steps of JA biosynthesis. Among all six 13‐lipoxygenase genes (*LOX7*, *LOX8*, *LOX9*, *LOX10*, *LOX11*, and *LOX13*) that enable the production of 12‐oxo‐phytodienoic acid (12‐OPDA) and its downstream JA synthesis (Figure 2a), only *LOX10* and *LOX11* were highly induced by *S. frugiperda* feeding (Figure 2b). In contrast, six 9‐LOX genes (*LOX1*, *LOX2*, *LOX3*, *LOX4*, *LOX5*, and *LOX6*) that serve in the production of 10‐oxo‐11‐phytodienoic acid (10‐OPDA, positional isomer of 12‐OPDA) and 10‐oxo‐11‐phytoenoic acid (10‐OPEA) were all highly induced after *S. frugiperda* infestation. Overall, the expression of 9‐lipoxygenases was induced to higher levels than 13‐lipoxygenases in shoot tissue in response to *S. frugiperda* feeding. In addition, all the transcripts of allene oxide synthase (*AOS*), allene oxide cyclase (*AOC*), oxo‐phytodienoate reductase (*OPR*), and jasmonate resistant (*JAR*) were upregulated upon *S. frugiperda* herbivory (Figure 2b). Belowground infestation by *D. v. virgifera* induced the expression of one 13‐LOX gene (*LOX10*) and six 9‐LOX genes (*LOX1*, *LOX2*, *LOX3*, *LOX4*, *LOX5*, and *LOX6*) and repressed the expression of *LOX7* and *LOX12* in maize roots (Figure 2c). Most of the transcripts of *AOS*, *AOC*, *JAR*, and, especially, *OPR* were upregulated in roots after *D. v. virgifera* infestation, while aboveground infestation by *S. frugiperda* barely modified the expression of JA‐related genes in roots (Figure 2c).

The biosynthesis of SA in plants is regulated by the isochorismate synthase (*ICS*) and phenylalanine ammonia‐lyase (*PAL*) pathways (Figure 3a). Between the two distinct pathways, only the expression of genes involved in the *PAL* pathway was clearly upregulated in shoots after *S. frugiperda* feeding (*PAL4*, *PAL5*, *PAL6*, *PAL7*, and *PAL8*; Figure 3b) or in roots after *D. v. virgifera* infestation (*PAL4*, *PAL7*, and *PAL8*; Figure 3c).

Several genes involved in ABA biosynthesis (*ZEP*, zeaxanthin epoxidase; *NCED*, 9‐cis‐epoxycarotenoid dioxygenase; *SDR*, short‐chain dehydrogenase/reductase; *AO*, aldehydeoxidase; Figure 4a) were upregulated in shoots after *S. frugiperda* herbivory, and the expression of *ZEPc3*, *ZEP1*, *NCED*, and *AO* was higher in *S. frugiperda*‐infested shoots compared with artificially damaged shoots. Moreover, the expression of *SDR* in shoots was also induced by belowground herbivore or artificial damage (Figure 4b). In roots, *D. v. virgifera* infestation highly induced the transcription of *ZEPc2*, *ZEP1*, and *NCED*, while mechanical damage in roots induced the transcription of *ZEPc1*, *ZEP1*, and *SDR* (Figure 4c).

*S. frugiperda* herbivory but not artificial damage induced genes involved in ethylene biosynthesis in maize shoot, but repressed the expression of ethylene insensitive 2 (*EIN2*), the central transducer of ethylene signal (Figure 5a,b). The expression of two 1‐aminocyclopropane‐1‐carboxylate oxidase (*ACO*) genes involved in ethylene synthesis was highly upregulated in roots after *D. v. virgifera* infestation, whereas the transcription of several ethylene biosynthesis genes was highly induced in response to artificial damage in roots (Figure 5c).

### 2.5 Benzoxazinoid biosynthesis‐related genes induced by belowground and aboveground insect herbivory

**FIGURE 6:**
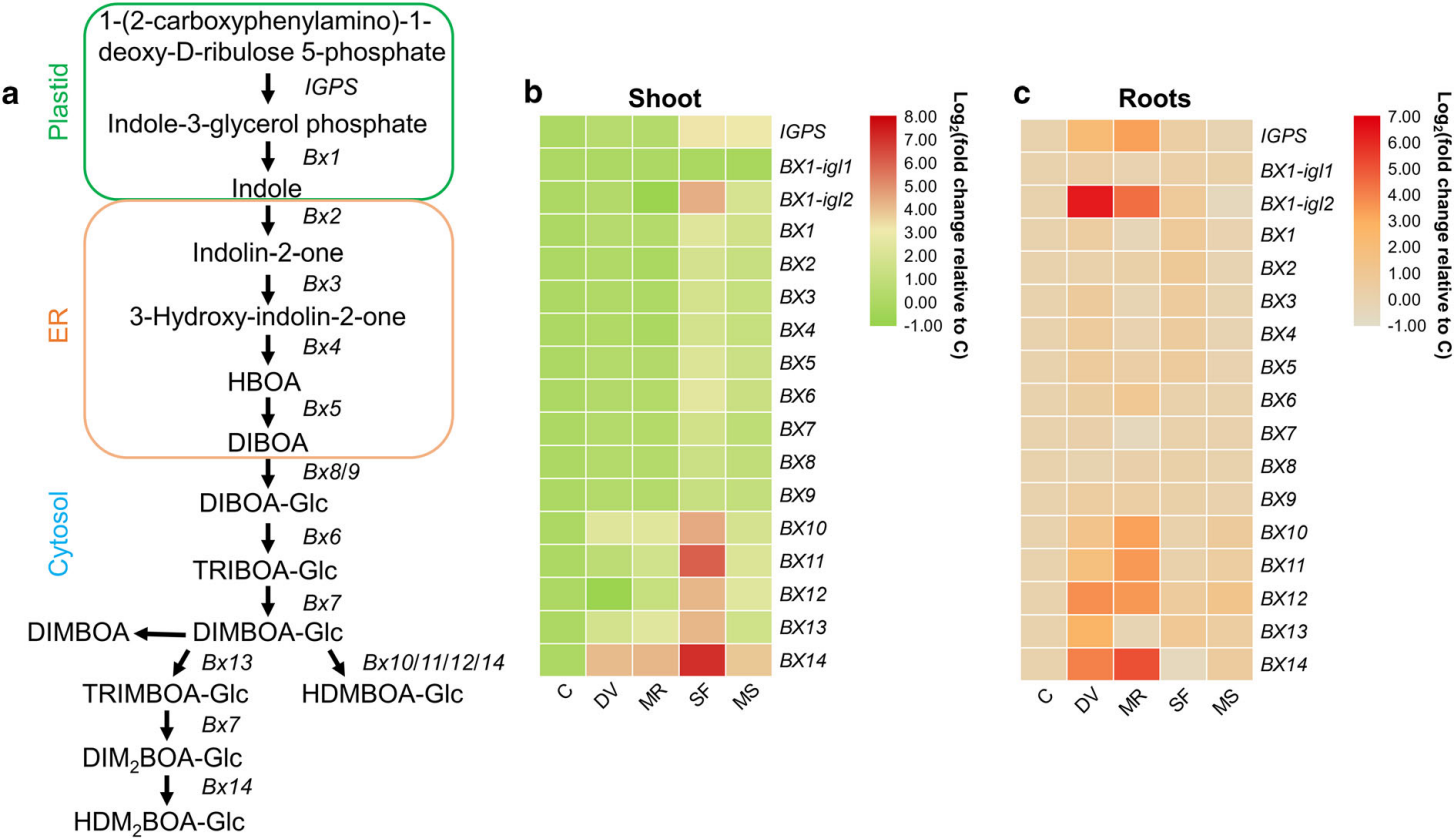
Effects of belowground and aboveground insect herbivory on benzoxazinoid biosynthesis pathway gene expression. (a) Schematic diagram of the benzoxazinoid biosynthesis pathway. ER, endoplasmic reticulum; *IGPS*, indole‐3‐glycerolphosphate synthase gene; HBOA, 2‐hydroxy‐1,4‐benzoxazin‐3‐one; DIBOA, 2,4‐dihydroxy‐1,4‐benzoxazin‐3‐one; DIBOA‐Glc, 2,4‐dihydroxy‐1,4‐benzoxazin‐3‐one β‐D‐glucopyranose; TRIBOA‐Glc, 2‐hydroxy‐1,4‐benzoxazin‐3‐one β‐D‐glucopyranose; DIMBOA‐Glc, 2,4‐dihydroxy‐7‐methoxy‐1,4‐benzoxazin‐3‐one β‐D‐glucopyranose; DIMBOA, 2,4‐dihydroxy‐7‐methoxy‐1,4‐benzoxazin‐3‐one; HDMBOA‐Glc, 2‐hydroxy‐4,7‐dimethoxy‐1,4‐benzoxazin‐3‐one β‐D‐glucopyranose; TRIMBOA‐Glc, 2‐2,4,7‐trihydroxy‐8‐methoxy‐1,4‐benzoxazin‐3‐one β‐D‐glucopyranose; DIM2BOA‐Glc, 4‐dihydroxy‐7,8‐dimethoxy‐1,4‐benzoxazin‐3‐one β‐D‐glucopyranose; HIDM2BOA‐Glc, 2–2‐hydroxy‐4,7,8‐trimethoxy‐1,4‐benzoxazin‐3‐one β‐D‐glucopyranose (modified from Tzin et al., 2017). (b and c) Heat map of benzoxazinoid biosynthesis‐related gene expression in maize shoot (b) and roots (c). Samples were collected from maize plants that were kept non‐manipulated (C, control) or after 72 h of belowground infestation by *Diabrotica virgifera virgifera* (DV), mechanical damage on root (MR), 72 h of aboveground infestation by *Spodoptera frugiperda* (SF), or mechanical damage on shoot (MS). Color coding represents the range of log_2_(fold change relative to control).

We compared the expression of several genes associated with benzoxazinoid biosynthesis (Figure 6a). Compared with artificial leaf damage, *S. frugiperda* feeding highly induced all genes involved in BX biosynthesis except *BX1‐igl1* (indole glycerol phosphate lyase) in shoot tissue. This was particularly the case for *BX1‐igl2*, which is potentially involved in indole production and several *BX* genes that are required for the synthesis of HDMBOA‐Glc (*BX10*, *BX11*, *BX12*, and *BX14*), TRIMBOA‐Glc (*BX13*) and HDM_2_BOA‐Glc (*BX14*) (Figure 6b). Moreover, belowground infestation by *D. v. virgifera* and artificial root damage significantly upregulated the expression of *BX10*, *BX13*, and *BX14* in shoot tissues (Figure 6b). Root herbivory by *D. v. virgifera* induced a similar expression pattern of *BX* genes in maize roots compared with that in leaf tissue after shoot herbivory. Furthermore, aboveground herbivory by *S. frugiperda* slightly upregulated several *BX* genes responsible for DIBOA (*BX1*, *BX2*, *BX3*, *BX4*, and *BX5*), HDMBOA‐Glc (*BX12*), and TRIMBOA‐Glc (*BX13*) synthesis in maize roots (Figure 6c).

### 2.6 Volatile terpene biosynthesis‐related genes induced by belowground and aboveground insect herbivory

**FIGURE 7:**
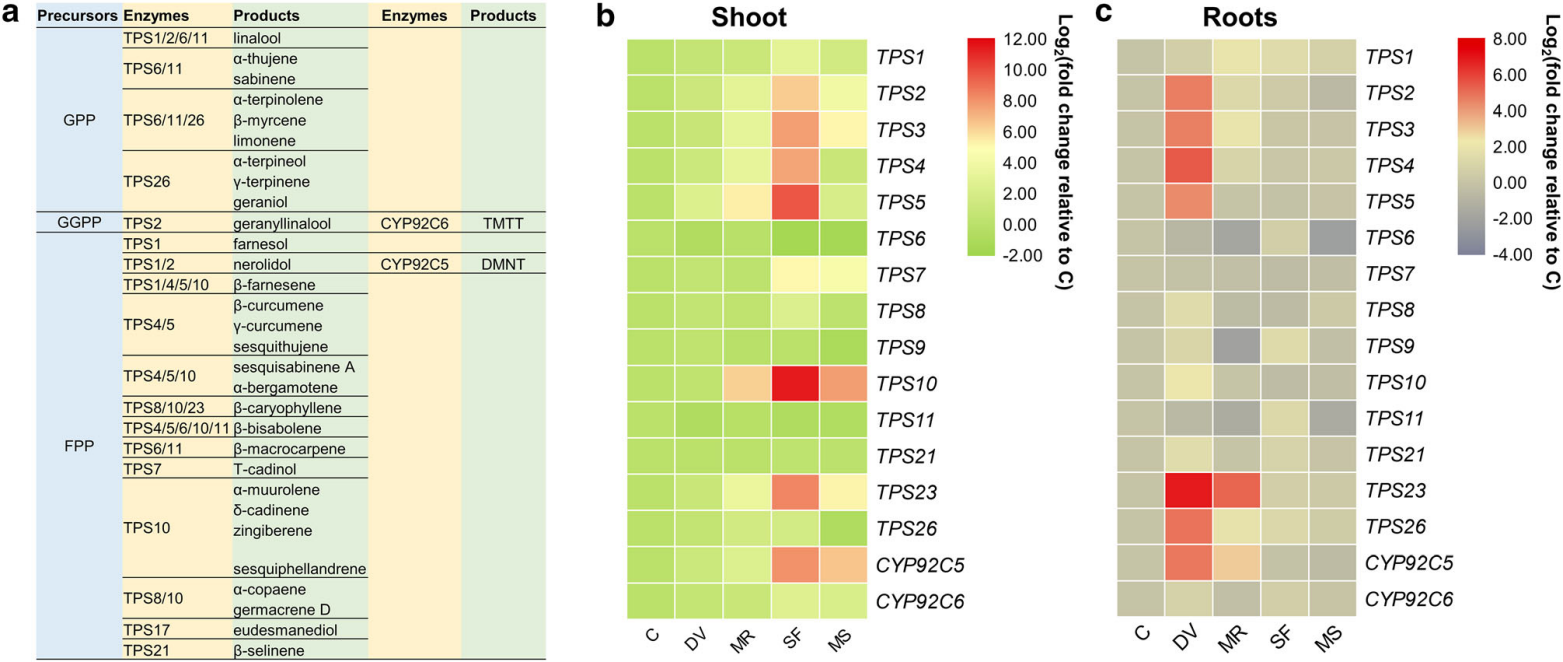
Effects of belowground and aboveground insect herbivory on volatile terpene biosynthesis gene expression. (a) Enzymes involved in the production of volatile terpenes in maize. GPP, geranyl diphosphate; GGPP, geranylgeranyl diphosphate; FPP, farnesyl diphosphate; TPS, terpene synthase; CYP92C5 and CYP92C6, cytochrome P450 monooxygenases; TMTT, (*E*,*E*)‐4,8,12‐trimethyltrideca‐1,3,7,11‐tetraene; DMNT, (*E*)‐3,8‐dimethyl‐1,4,7‐nonatriene (Block et al., 2019). (b) and (c) Heat map of volatile terpene biosynthesis gene expression in maize shoot (b) and roots (c). Samples were collected from maize plants that were kept non‐manipulated (C, control) or after 72 h of belowground infestation by *Diabrotica virgifera virgifera* (DV), mechanical damage on root (MR), 72 h of aboveground infestation by *Spodoptera frugiperda* (SF), or mechanical damage on shoot (MS). Color coding represents the range of log_2_(fold change relative to control).

Lastly, we analyzed the expression of genes coding for terpene synthases (TPS) (Figure 7), which are enzymes that control the synthesis of herbivory‐induced volatile terpenes that may function as indirect defenses in plants (Block et al., 2019). In maize shoot, all *TPS* genes except *TPS6*, *TPS9*, *TPS11*, and *TPS21* were highly induced by *S. frugiperda* feeding. *S. frugiperda* herbivory also upregulated two cytochrome P450 monooxygenases, *CYP92C5* and *CYP92C6*, which respectively catalyze transformation of (*E*)‐nerolidol and (*E*,*E*)‐geranyllinalool to (3*E*)‐4,8‐dimethyl‐1,3,7‐nonatriene (DMNT) and (*E*,*E*)‐4,8,12‐trimethyltrideca‐1,3,7,11‐tetraene (TMTT) (Richter et al., 2016) (Figure 7b). In addition, artificial root damage and root herbivory by *D. v. virgifera* induced several volatile terpene biosynthesis‐related genes in shoot tissue but to a much lesser extent than aboveground herbivory and damage (Figure 7b). In maize roots, infestation by *D. v. virgifera* more strongly induced volatile‐related genes (especially *TPS2*, *TPS3*, *TPS4*, *TPS5*, *TPS23*, *TPS26*, and *CYP92C5*) than artificial root damage. Aboveground herbivory by *S. frugiperda* also slightly upregulated the expression of *TPS1*, *TPS9*, *TPS11*, and *TPS26* in roots (Figure 7c).

## 3 DISCUSSION

By analyzing changes in the maize transcriptome, we revealed defense responses of plants to two well‐adapted insect herbivores, *D. v. virgifera* and *S. frugiperda*. Artificial root and leaf damage were used for comparison to determine the specific transcriptomic responses of maize plants to these specialized insects. The results reveal that belowground infestation by *D. v. virgifera* larvae and aboveground feeding by *S. frugiperda* caterpillar trigger local and systemic transcriptome changes that differ in various ways from responses to artificial damage. *D. v. virgifera* and *S. frugiperda* caused more upregulated DEGs than downregulated DEGs in the specific tissue they fed on, root and shoot, respectively (Figure 1c,d). This is similar to transcriptome responses reported for herbivory by the beet armyworm *Spodoptera exigua* (Tzin et al., 2017) and Asian corn borer *Ostrinia furnacalis* (Guo et al., 2019) and implies that the maize plants respond not just to the mechanical damage caused by these insects, but also to possible elicitors and effectors that are introduced into the plants while they are feeding. Thus far, several potent elicitors from OS of *Spodoptera* caterpillars such as volicitin and inceptin have been identified (Alborn et al., 1997; Schmelz et al., 2006; Turlings et al., 2000) and the mechanisms underlying elicitor‐mediated defense responses have been extensively studied (Erb, Meldau, et al., 2012). Much less is known about effectors and their role in modulating plant defenses. Moreover, the identity and mode of action of root herbivore elicitor/effector remain unclear (Johnson et al., 2016).

Although root herbivore‐induced leaf resistance has been extensively studied for maize (Erb, Flors, et al., 2009; Erb, Gordon‐Weeks, et al., 2009; Erb, Köllner, et al., 2011), little is known about the molecular mechanism underlying root‐to‐shoot signaling. We found that belowground wounding by the root herbivore and artificial root damage both also markedly changed the shoot transcriptome (Figure 1c), offering a dataset to help understand how root herbivory systemically affects defenses in maize plants. Even less is known about the impact of aboveground infestation on root defense, but we know that if *S. frugiperda* attacks maize before *D. virgifera*, the root herbivore's performance is negatively affected (Erb, Robert, et al., 2011). Similarly, leaf attack by diamondback moth caterpillars *Plutella xylostella* strongly reduces the performance of cabbage root fly larvae *Delia radicum* on cabbage plants *Brassica oleracea* (Karssemeijer et al., 2020). Our transcriptome data suggest that aboveground wounding (insect or artificial) causes only minor changes in maize roots (Figure 1d). This may be due to a transient transcriptomic change in the roots that only occurs early during leaf‐herbivory and therefore could not be detected in our 3‐day experiment. It is also possible that a minor transcription change in the roots upon leaf herbivory is enough to trigger an effective root defense.

Feeding by *S. frugiperda* caterpillars was found to cause significant changes in the regulation of primary and secondary metabolism pathways in maize shoots. Transcriptomic changes in the biosynthesis of phenylpropanoid, flavonoid, benzoxazinoid, and metabolisms related to production of phytohormones and volatiles (Figure S2A) indicate their role in defense against *S. frugiperda* herbivory. The much stronger responses caused by *S. frugiperda* herbivory than by artificial leaf damage in the photosynthesis pathway (Figure S2B) might be explained by a compensatory growth response to consumption of leaf tissue by *S. frugiperda*. It appears that plants can differentiate between herbivory and mere mechanical damage and regulate their photosynthesis in accordance with growth‐defense trade‐offs (Visakorpi et al., 2018). Leaf damage by *S. frugiperda* also induced phenylpropanoid, flavonoid and benzoxazinoid biosynthesis in root tissues (Figure S2C), confirming that aboveground herbivory may affect root‐herbivore performance, as previously shown in insect performance assays (Erb, Robert, et al., 2011).

It is known that maize plants, in order to cope with *S. frugiperda* attack, activate the expression of genes involved in direct defense such as genes encoding protease and proteinase inhibitors (Pechan et al., 2002; Ton et al., 2007) and indirect defense such as genes related to volatile emissions (Köllner et al., 2008; Schnee et al., 2006) (Figure S3A). Interestingly, our results also show that *S. frugiperda* feeding suppresses the expression of several candidate stress response‐related genes, such as MYB20 (Zm00001d002545) that is involved in secondary cell wall formation (Geng et al., 2020), MYB111 (Zm00001d026017) that is involved in regulating flavonoid biosynthesis (Li et al., 2019; Stracke et al., 2010), and indole‐2‐monooxygenase‐like (Zm00001d035178) that is putatively involved in DIBOA‐glucoside biosynthesis. We show that genes associated with primary metabolism, like monooxygenase/oxidoreductase (Zm00001d021444) that is involved in auxin biosynthesis and transcription factor LUX (*LUX ARRHYTHMO*) (Zm00001d041960) necessary for circadian rhythms (Gil & Park, 2019), are also repressed by *S. frugiperda* caterpillar feeding (Figure S3B). Evidently, maize plants strongly alter primary and secondary metabolism in response to *S. frugiperda* herbivory, but *S. frugiperda* caterpillars may, as a counteradaptation, also suppress maize defense (De Lange et al., 2020).

Apart from changes in the regulation of primary metabolism, belowground herbivory by *D. virgifera* larvae was also found to modify secondary metabolism pathways such as the biosynthesis of phenylpropanoids and monoterpenoids in maize roots (Figure S4A). The difference in pathway enrichment between root herbivory and artificial root damage (Figure S4B) suggests that maize plants distinguish between root herbivore and artificial wounding and reprogram their transcriptome accordingly. Importantly, maize also adjusts its DNA replication aboveground in response to root attack by *D. virgifera* (Figure S4C), which probably affects the growth and development of the shoot (Castellano et al., 2004). Interestingly, a putative methyl salicylate biosynthesis‐related gene (benzenoid carboxyl methyltransferase *omt7*, Zm00001d052828) is not expressed in leaves of maize after leaf herbivory (Köllner et al., 2010) but can be induced in roots by drought stress (Zheng et al., 2020). We found that this gene is also induced by *D. virgifera* feeding (Figure S5A), suggesting that *D. virgifera* attack and drought stress both induce root‐specific methyl salicylate. Another interesting gene is the one coding for anthranilic acid methyltransferase1 (*aamt1*, Zm00001d044762) responsible for the production of methyl anthranilate (Köllner et al., 2010), a repellent for *D. virgifera* (Bernklau et al., 2016). It was induced by both types of root damage (Figure S5A). In contrast, the expression of several genes involved in the regulation of plant defense and resistance in shoots, for example, two putative LRR protein genes (Bianchet et al., 2019; Ye et al., 2020) and a cysteine proteinase inhibitor gene (Ton et al., 2007), were downregulated in response to *D. virgifera* feeding (Figure S5B and Data S8). The potential role of these genes in belowground plant‐insect interactions still needs to be elucidated. Surprisingly, the transcription levels of several photosynthetic genes were also repressed in herbivore infested‐roots, a nonphotosynthetic organ, but their expression levels are much lower than that in leaves (Figure S5B, Data S1, and Data S8). Previous research showed that the suppression of photosynthetic gene expression is required for sustained root growth in *Arabidopsis* under phosphate deficiency (Kang et al., 2014). Possibly, herbivore infested‐roots suffer from phosphate deficiency caused by root damage; it is also possible that biotic stress in general reduces the expression of photosynthetic genes to promote root growth to compensate for root consumption by larvae. In summary, it appears that maize plants not only switch on their defenses in response to *D. virgifera* infestation but also adjust growth and development in both shoot and roots, preparing for tissue regeneration.

The phytohormone network that comprises JA, SA, ABA, and ET signaling is highly important in regulating plant direct and indirect defenses against insects (Erb, Meldau, et al., 2012; Johnson et al., 2016; Wu & Baldwin, 2010). The essential role of JA signaling in the activation of local and systemic defense against chewing insect attack is well studied (Lortzing & Steppuhn, 2016; Lu et al., 2015). The start of JA biosynthesis is catalyzed by 13‐LOX from α‐linolenic acid before being converted to 12‐OPDA by AOS and AOC (Lu et al., 2015; Figure 2a). A similar metabolic branch is catalyzed by 9‐LOX from linolenic and linoleic acid to produce 10‐OPDA and 10‐OPEA, respectively (Tzin et al., 2017). Both 10‐OPDA and 10‐OPEA display phytotoxicity, and local production of 10‐OPEA and associated death acids (DAs) in maize induced by fungal southern leaf blight (*Cochliobolus heterostrophus*) act as a phytoalexin by suppressing the growth of fungi and herbivores (Christensen et al., 2015). A total of six potential 13‐lipoxygenase coding genes and seven candidate 9‐lipoxygenase coding genes have been predicted for the sequenced B73 maize genome (Woldemariam et al., 2018). Among these genes, *LOX10* has been confirmed to mediate the production of green leaf volatiles, jasmonates, and herbivore‐induced plant volatiles in maize plants (Christensen et al., 2013). In our study, two 13‐*LOX* genes (*LOX10* and *LOX11*) and all 9‐*LOX* genes (especially *LOX1*, *LOX2*, *LOX3*, and *LOX5*) except for *LOX12* were highly induced in the shoot upon *S. frugiperda* attack. In general, the expression of 9‐*LOX* genes was more strongly induced than 13‐*LOX* genes (Figure 2b), which is largely consistent with the reported expression patterns of *LOX* genes in maize leaves fed upon by the Asian corn borer *Ostrinia furnacalis* (Guo et al., 2019) and the beet armyworm *Spodoptera exigua* (Tzin et al., 2017), suggesting that the initiation of JA signaling in maize is similar in response to different chewing herbivores. Considering the strong expression of 9‐*LOX* genes in maize leaves infested by lepidopteran herbivores as well as the local phytoalexin activity of DAs produced through 9‐LOX catalyzation, the activity of 9‐LOX might be involved in the direct defense of maize against caterpillar attack. *LOX10* appears to be only slightly upregulated by *S. exigua* feeding (Tzin et al., 2017), whereas it is relatively strongly induced by *S. frugiperda* (Figure 2b) and *O. furnacalis* (Guo et al., 2019). This may reflect a difference between herbivore species, but may also be due to the use of different numbers of caterpillars or different maize lines. All the other genes involved in subsequent steps of JA biosynthesis in maize shoot were found to be upregulated by *S. frugiperda* feeding, especially *AOS2*, *OPR1*, and *OPR2* (Figure 2b), possibly reflecting the respective importance of these genes in the defense response to caterpillar attack. Another important defense gene, *JAR1*, mediates the production of jasmonoyl‐isoleucine conjugate (JA‐Ile), the active form of JA (Koo & Howe, 2012). The expression of *JAR1a* rather than *JAR1b* is highly induced by caterpillar attack on maize leaves (Guo et al., 2019; Tzin et al., 2017), and a similar increase in *JAR* transcription level was observed in our study (Figure 2b), further suggesting the importance of maize *JAR1a* in the biosynthesis of JA‐Ile.

In accordance with the assumed role of JA signaling being involved in the local defense of plant roots against belowground herbivores (Lu et al., 2015), we found that a group of JA‐related genes is induced by *D. v. virgifera* feeding on maize roots (Figure 2c). However, in comparison with the leaf response to aboveground herbivore feeding, maize roots increased their expression levels of JA‐related genes to a lesser extent in response to belowground feeding (Figure 2 and Data S9). Similarly, JA levels in maize roots were found to only increase about two fold upon *D. v. virgifera* attack (Erb, Flors, et al., 2009), which is considerably less compared with JA increases in leaves in response to caterpillar feeding (Schmelz et al., 2003). This is perhaps due to the different sensitivity of JA signaling in roots and shoot to herbivores. In a previous study, short‐term JA signaling was differently induced (within 24 h) by belowground herbivore attack and artificial root damage, but neither the content of JA nor the expression levels of *LOX* and *JAR1* showed pronounced differences in roots after 24 h of herbivory or mechanical damage (Lu et al., 2015). Whether the maize roots can specifically recognize herbivores as is known for shoots (Chuang et al., 2014; Qi et al., 2016; Schmelz et al., 2009) still needs to be explored. We also found that belowground herbivory slightly induced several JA‐related genes in the shoot, whereas aboveground herbivory hardly changed JA signaling in roots (Figure 2 and Data S9), suggesting that aboveground JA signaling is mainly responsible for local defense, whereas root JA signaling might be involved in root‐to‐shoot communication. This appears to also be the case in *Arabidopsis thaliana*, where early systemic JA responses in the shoot have been found to be even higher compared with the local responses in roots to artificial wounding (Hasegawa et al., 2011).

SA is another important phytohormone for plant immunity that functions in basal defense and systemic acquired resistance (SAR) (Huang et al., 2020). The biosynthesis of SA in plants follows two independent pathways, ICS and PAL (Dempsey et al., 2011; Huang et al., 2020). We found that upon aboveground and belowground herbivore attack, a number of genes involved in PAL but not ICS pathway are induced in maize (Figure 3). A similar SA‐related gene expression pattern has been reported for maize leaves after *O. furnacalis* infestation (Guo et al., 2019). OS application of *Mythimna separata* to maize leaf wound sites also strongly elicits SA accumulation (Qi et al., 2016). However, aboveground herbivory by *Spodoptera littoralis* and *O. furnacalis*, or belowground herbivory by *D. v. virgifera*, do not increase SA concentration in maize leaf and roots, respectively (Erb, Flors, et al., 2009; Guo et al., 2019). Similarly, belowground attack by cucumber beetle *Diabrotica balteata* and rice water weevil *Lissorhoptrus oryzophilus* do not increase the SA content in rice roots (Lu et al., 2015). PAL, which enables the production of cinnamic acid and its downstream phenolic products caffeic acid and ferulic acid, is involved in the phenylpropanoid metabolism pathway. In maize, the levels of caffeic acid and ferulic acid have been reported to increase after 6 h infestation by *S. exigua* and to decrease after 24 h, which might be because these phenylpropanoids serve as substrate/precursors for the biosynthesis of other defensive compounds (Tzin et al., 2017). Instead of activating SA signaling, maize plants might mobilize phenylpropanoid metabolism by increasing the expression of *PAL* genes to accelerate downstream defensive metabolite accumulation, thereby protecting themselves against shoot and root attacks. Hydroxycinnamic acid amides form a diverse group of specialized phenylpropanoid metabolites in many plants. The abundance of several hydroxycinnamic acid amide derivatives such as coumaroyltyramine, coumaroyltryptamine, and feruloyltyramine is highly increased in maize leaves after *S. littoralis* attack (Marti et al., 2013). The importance of these metabolites in plant defense still needs to be examined.

The regulator function of ABA and ET in plant defense and resistance is well documented (Broekgaarden et al., 2015; Erb & Reymond, 2019; Olds et al., 2018; Vos et al., 2013). For instance, ABA‐deficient *Arabidopsis* mutant plants are more susceptible to *S. littoralis* (Bodenhausen & Reymond, 2007). Here, maize plants increased the expression of a series of ABA‐related genes in shoot and roots in response to herbivory by *S. frugiperda* and *D. v. virgifera*, respectively (Figure 4). This is consistent with previous studies of ABA induction in maize plants upon *O. furnacalis* (Guo et al., 2019) and *D. v. virgifera* (Erb, Flors, et al., 2009) attack. However, *S. littoralis* infestation does not increase the ABA level in maize shoot (Erb, Flors, et al., 2009), and in rice roots, the biosynthesis of ABA is not induced by belowground *D. balteata* and *L. oryzophilus* attack (Lu et al., 2015). Considering the crosstalk between ABA and JA signaling and the role of ABA in drought stress response, it is expected that the ABA pathway is involved in systemic defenses against herbivores (Erb, Flors, et al., 2009; Erb, Köllner, et al., 2011; Wang et al., 2018). A previous study showed that exogenous application of ABA on maize root boosts aboveground defense (Erb, Gordon‐Weeks, et al., 2009). We found a few ABA biosynthesis‐related genes to be induced in both shoot and root in response to belowground and aboveground herbivory, respectively (Figure 4). Notably, even though artificial leaf damage and aboveground *S. frugiperda* herbivory increase the transcription level of *ZEP1* and *ZEP2*, respectively, in maize roots, the expression of *NCED* was found to be repressed (Figure 4c), which might lead to the homeostasis of ABA levels in roots. Taken together, the results imply that ABA signaling is probably not only involved in maize local defenses against *S. frugiperda* and *D. v. virgifera* herbivory, but also partly responsible for systemic defenses against herbivores.

The effect of ethylene (ET) on plant defense is variable. In maize, it positively regulates resistance to *S. frugiperda* in Mp708, an insect‐resistant maize inbred line, but not in Tx610, a susceptible maize line (Harfouche et al., 2006). The transcription of a rice ET biosynthesis‐related gene 1‐aminocyclopropane‐1‐carboxylic acid synthase (*OsACS2*) can be induced by wounding and herbivory, and silencing of *OsACS2* has been shown to suppress ET production and reduce resistance to a chewing herbivore, the striped stem borer *Chilo suppressalis* (Lu et al., 2014). Partially consistent with this result, simulated caterpillar herbivory (artificial damage plus the application of oral secretion from *M. separata*), in comparison with mechanical wounding only, highly increases the concentration of ET in maize leaf tissue (Qi et al., 2016). Similarly, in our study, the transcription of four ET biosynthesis‐related genes in maize shoots was induced by *S. frugiperda* feeding but not mechanical wounding (Figure 5b). Compared with wild type plants, *Arabidopsis* ET insensitive mutant *ein2‐1* is more resistant to generalist *S. littoralis*, but not to specialist diamondback moth *Plutella xylostella*. In addition, exogenous application of ET by treating the plant with ethephon (2‐chloroethanephosphonic acid) leads to enhanced resistance to *S. littoralis* (Stotz et al., 2000). Furthermore, in *Arabidopsis thaliana*, a double mutant of ET‐stabilized transcription factor ET insensitive3 and ET insensitive3‐like 1 (*ein3 eil1*) shows enhanced defense against *S. exigua*, and this is probably due to the JA and ET signaling antagonism in regulating plant wounding response and defense against insect attack (Song et al., 2014). Interestingly, the expression of *EIN2*, the central component of the ET signaling pathway, was repressed in maize shoots in response to *S. frugiperda* attack (Figure 5b). Taken together, our data suggest that the biosynthesis of ET in maize shoot is activated in response to *S. frugiperda* attack, while downstream the ET signaling pathway might be suppressed by JA‐ET antagonism in order to protect maize plants against *S. frugiperda*. In contrast to aboveground herbivory, both root wounding by *D. v. virgifera* feeding and artificial root damage increased the expression of several genes involved in ET signaling (Figure 5c). This is different in rice, where the concentration of ET is not increased in response to belowground herbivory by *D. balteata* (Lu et al., 2015). In summary, ET appears essential for modulating plant defenses against herbivores, but these defenses are plant species‐, genotype‐, tissue‐, and herbivore‐specific.

In addition to these typical plant defense hormones, we also targeted benzoxazinoids. These defense metabolites occur in many monocots, including maize, and are effective in providing resistance against insect herbivores (Tzin et al., 2017). However, well‐adapted herbivores such as *D. v. virgifera* and *S. frugiperda* have been shown to tolerate high concentrations of benzoxazinoids and even use benzoxazinoids as foraging cues (Köhler et al., 2015; Robert et al., 2012). In maize shoot, aboveground herbivory by *S. frugiperda* caused a significantly higher expression of *BX* genes compared with artificial leaf damage, whereas belowground herbivory and artificial root damage resulted in a similar increase of *BX* genes expression pattern in maize roots (Figure 6). This was consistent with the JA‐related gene expression pattern in maize shoot and roots upon herbivory and mechanical damage (Figure 2). JA induces the production of benzoxazinoids in maize (Tzin et al., 2017), and this might explain the similarity between the expression pattern of JA‐ and benzoxazinoid biosynthesis‐related genes in maize roots upon herbivory and mechanical damage. Furthermore, compared with the minor impact that aboveground *S. frugiperda* herbivory and artificial shoot damage had on root gene expression, belowground *D. v. virgifera* feeding and artificial root damage had a much stronger effect on the expression in the shoots of a series of downstream benzoxazinoid biosynthesis‐related genes (Figure 6). This implies that root herbivory and artificial root damage can induce shoot defense and resistance against leaf herbivores, and root‐to‐shoot JA signaling might be involved in mediating this systemic defense in maize plants.

Plants have also evolved the ability to attract predators and parasitoids with herbivore‐induced plant volatiles (HIPVs) (Dicke & Baldwin, 2010; Turlings & Erb, 2018). Volatile terpenoids such as (*E*)‐β‐caryophyllene (Rasmann et al., 2005; Xiao et al., 2012), DMNT, and TMTT (Tamiru et al., 2011) play a critical role in this indirect defense. Herbivore‐induced terpene production is regulated by the expression of genes of the TPS family (Block et al., 2019). *TPS2* and two cytochrome P450 enzyme coding genes, *CYP92C5* and *CYP92C6*, are responsible for the production of DMNT and TMTT in maize (Richter et al., 2016). In this study, we confirm that *S. frugiperda* and *D. v. virgifera* attack increases the expression of a number of *TPS* genes in shoot and roots and more so than artificial damage (Figure 7). These *TPS* genes are involved in the biosynthesis of the major volatile terpenes emitted by herbivore‐infested maize plants such as nerolidol (*TPS1* and *TPS2*), (*E*)‐β‐caryophyllene (*TPS8*, *TPS10*, and *TPS23*), (*E*)‐α‐bergamotene (*TPS4*, *TPS5*, and *TPS10*), (*E*)‐β‐farnesene (*TPS1*, *TPS4*, *TPS5*, and *TPS10*), and DMNT (*TPS2* and *CYP92C5*) (De Lange et al., 2020). *D. v. virgifera* herbivory and artificial root damage also slightly but significantly induced the expression of a few *TPS* genes in maize shoots, and *S. frugiperda* attack had the same effect on maize roots (Figure 7). Hence, our results confirm that maize plants increase their volatile terpenoid biosynthesis in response to aboveground and belowground herbivory.

In this study, we evaluated the transcriptomic changes in maize plants upon aboveground and belowground attack by the specialized herbivores *S. frugiperda* and *D. v. virgifera* and compare these changes to those triggered by artificially damage. The comprehensive assessment of local and systemic transcriptomic changes of herbivore‐infested plants provides new insight into the molecular mechanism underlying induced resistance in maize against leaf‐ and root‐herbivores, as well as into the plant's growth‐defense balance. In addition, the presented data can serve as a basis for further exploration of novel crop protection strategies that modify and exploit herbivore induced defenses.

## 4 METHODS

### 4.1 Plants and herbivores

Maize seedlings (*Zea mays* var. Delprim) were grown individually in plastic pots (height 10 cm; diameter 4 cm) using a mixture of commercial potting soil (Einheitserde Classic, Gebrüder Patzer GmbH & Co. KG, Germany) and sand (Sable Capito 1–4 mm, Landi, Dotzigen, Switzerland) in equal proportion (1:1; v/v) under controlled conditions (28 ± 2°C; 60% relative humidity; 16‐/10‐h light/dark photoperiod) in the greenhouse. Two insect species were used for the experiments. The leaf herbivore *Spodoptera frugiperda* (JE Smith) (Lepidoptera: Noctuidae) and the root herbivore *Diabrotica virgifera virgifera* LeConte (Coleoptera: Chrysomelidae) were obtained from laboratory colonies at the University of Neuchâtel. The larvae of *S. frugiperda* were reared on artificial diet as described by Turlings et al. (2004). The larvae of *D. v. virgifera* were maintained on freshly germinated maize roots as described by Erb, Robert, et al. (2011).

### 4.2 Mechanical damage and herbivory treatments

Twenty‐day‐old maize plants were used for the experiments. We randomly assigned 12 plants to each of the following five treatments: roots infested by (1) *D. v. virgifera* or (2) mechanically damaged; shoots infested by (3) *S. frugiperda* or (4) mechanically damaged and (5) uninfested controls (hereafter identified as treatments DV, MR, SF, MS, and C, respectively). For DV treatment, five second‐instar larvae of *D. v. virgifera* were released onto the soil surface around the stem of maize plant to infest the roots. After 72 h infestation, the whole roots were harvested. The larvae were removed from the roots immediately during root tissue harvest. For MR treatment, the roots were mechanically damaged by stabbing with a metal corkborer (diameter, 7 mm) at a depth of approximately 5 cm into the soil three times daily for 3 days based on the methods from Rasmann et al. (2005). For SF treatment, three newly molted third‐instar larvae of *S. frugiperda* were caged on a maize leaf using a small clip cage and allowed to feed for 72 h. The cage was moved to an intact leaf area three times per day. For MS treatment, we punched an area of approximately 2 × 10 mm^2^ with forceps on both sides of the central vein of the third and fourth leaf. This was repeated three times daily for 3 days and created a wounded leaf area of approximately 2 × 6 cm^2^ every day. The whole shoots and roots were harvested and flash‐frozen in liquid nitrogen at 72 h after treatment.

### 4.3 Library preparation and transcriptome sequencing

Tissue from three individual maize seedlings was combined into one experimental replicate, and four replicates were prepared for each treatment. A total amount of 1 μg RNA per sample was used for library construction. Sequencing libraries were generated using NEBNext® Ultra™ RNA Library Prep Kit for Illumina® (NEB, USA) following manufacturer's recommendations and index codes were added to attribute sequences to each sample. The PCR products were purified (AMPure XP system), and library quality was assessed on an Agilent 2100 (Agilent Technologies, Palo Alto, CA, USA). The clustering of the index‐coded samples was performed on a cBot Cluster Generation System using PE Cluster Kit cBot‐HS (Illumina) according to the manufacturer's instructions. After cluster generation, the library preparations were sequenced on an Illumina HiSeq 4000 platform and paired‐end reads (2 × 150 bp) were generated.

### 4.4 RNA‐seq data analysis

Paired‐end clean reads were mapped to the maize reference genome (B73 RefGen_v4) (Jiao et al., 2017) using HISAT2 v2.0.5 program (Kim et al., 2015) with default parameters. The expression levels of genes were analyzed by using HTSeq v0.6.1 software (Anders et al., 2015) with union mode and were calculated as fragments per kilobase of transcript per million fragments mapped (FPKM). Differentially expressed genes (DEGs) between different experimental treatments were filtered by using DESeq2 R package v1.20.0 (Love et al., 2014) with false discovery rate (FDR) < .05 (Benjamini & Hochberg, 1995) and an absolute value of log_2_‐transformed fold change (treatment/control) > 1. Pathway enrichment of KEGG (Kyoto Encyclopedia of Genes and Genomes) was analyzed by using KOBAS v3.0 (Xie et al., 2011) (adjusted *P* < .05 were considered significantly enriched). Plant responses in root and shoot samples elicited by the leaf‐ and root‐feeding herbivores were compared with those obtained by artificial shoot and root damage, and samples from seedlings that were kept non‐manipulated served as control. We refer to local plant responses for tissue that was directly infested with root or shoot herbivores, and systemic plant responses for roots or shoots that were not infested but were sampled from a plant damaged in the opposite tissue.

## CONFLICT OF INTEREST

The authors declare no conflict of interest.

## AUTHOR CONTRIBUTIONS

W.Y. and T.C.J.T. designed the research. W.Y. performed the experiments. W.Y., M.E., T.C.J.T., C.B.S., and T.D. advised on the experimental design and wrote and revised the manuscript.

## Supporting information


**Data S1.** Genes detected in all samples. Gene expression levels were shown by FPKMs. Maize shoot (S) and roots (R) were harvested from seedlings that were kept non‐manipulated (C, control) or treated with belowground infestation by *D. v. virgifera* (DV), mechanical wounding on root (MR), aboveground infestation by *S. frugiperda* (SF), or mechanical wounding on shoot (MS); NA: no annotation.

**Data S2** All differentially expressed genes (DEGs) in maize shoot and roots induced by *D. v. virgifera* infestation with a cut‐off of two‐fold change relative to the control. FC: fold change; NA: no annotation.

**Data S3** All DEGs in maize shoot and roots induced by mechanical root damage with a cut‐off of two‐fold change relative to the control. FC: fold change; NA: no annotation.

**Data S4** All differentially expressed genes (DEGs) in maize shoot and roots induced by *S.frugiperda* infestation with a cut‐off of two‐fold change relative to the control. FC: fold change; NA: no annotation.

**Data S5** All DEGs in maize shoot and roots induced by mechanical shoot damage with a cut‐off of two‐fold change relative to the control. FC: fold change; NA: no annotation.

**Data S6** KEGG pathway enrichment analysis of DEGs in the transcriptome of maize induced by different treatments.

**Data S7** The top 60 DEGs in maize shoot induced by *S. frugiperda* infestation. FC: fold change; NA: no annotation.

**Data S8** The top 60 DEGs in maize roots induced by *D. v. virgifera* infestation. FC: fold change; NA: no annotation.

**Data S9** The gene expression pattern of phytohormones, benzoxazinoids and terpene volatiles.

**Figure S1** Expression levels and correlations for maize genes from qRT‐PCR (left) and RNA‐seq gene expression data (right). The transcript levels (mean + SE, *n* = 4) of eight genes in shoots of maize seedlings are shown after 72 h of belowground infestation by *Diabrotica virgifera virgifera* (DV), aboveground infestation by *Spodoptera frugiperda* (SF), application of root (MR) or shoot mechanical damage (MS). Non‐treated seedlings served as controls (C). The following genes were measured: *BX14* (benzoxazinone synthesis14, Zm00001d004921), *ZRP4‐like* (O‐methyltransferase, Zm00001d038703), *PR5* (pathogenesis‐related protein5, Zm00001d031158), *PR10* (pathogenesis related protein10, Zm00001d028816), *LOX3* (lipoxygenase, Zm00001d033623), *PPO* (polyphenol oxidase, Zm00001d000001), *BBTI13* (Bowman‐Birk type trypsin inhibitor, Zm00001d048660), *CLH* (chlorophyllase1, Zm00001d019758). For qRT‐PCR data, fold‐change of gene expression level was calculated using the 2^−ΔΔCT^ method. The results (threshold cycle values) of the qRT‐PCR assays were normalized to the expression level of *ZmCUL* (cullin, Zm00001d024855). For RNA‐seq data, gene expression levels were calculated as FPKM (fragments per kilobase of transcript per million fragments mapped).
**Figure S2** KEGG pathway enrichment analysis of differentially expressed genes (DEGs) in maize induced by *S. frugiperda* herbivory. (**A**) The top 20 enriched KEGG pathways in maize shoot between *S. frugiperda* herbivory (SF) and non‐manipulated control (C). (**B**) The top 20 enriched KEGG pathways in maize shoot between *S. frugiperda* herbivory and artificial shoot damage (MS). (**C**) The enriched KEGG pathways in roots of maize seedlings between *S. frugiperda* herbivory and control. Enrichment scores are shown as ‐log10(adjusted *P* value). Number of DEGs involved in each pathway are shown above the bar.
**Figure S3** Heatmap of the relative expression levels (fold change after log2 transformation) of the 60 most up‐ (**A**) and down‐regulated (**B**) genes in maize shoot induced by *Spodoptera frugiperda* feeding. Samples were collected from maize plants that were kept non‐manipulated (C, control) or after 72 h of belowground (DV) or aboveground infestation (SF), or mechanical damage on roots (MR) or shoot (MS). Color coding represents the range of log2(fold change relative to control). Color bar after gene ID and pie chart under heatmap showing the potential gene function. (**A**) The top 60 most up‐regulated genes in shoots induced by *S. frugiperda* feeding, included 25 defense response‐related genes (e.g. proteinase inhibitor, β‐glucosidase, O‐methyltransferase, and genes involved in ethylene‐, benzoxazinone‐, flavonoid‐synthesis) and 11 volatile biosynthesis‐related genes (e.g. terpene synthase, germacrene A synthase, dimethylnonatriene synthase, linalool synthase). Several genes, such as Bowman‐Birk type trypsin inhibitor (Zm00001d048660), O‐methyltransferase *ZRP4* (Zm00001d038703), ethylene biosynthesis‐related gene 1‐aminocyclopropane‐1‐carboxylate oxidase 3 (Zm00001d024852), and dirigent protein (Zm00001d004220) were induced by both shoot and root damage, implying their potential role in the systemic defense response to below‐ and aboveground herbivory. A total of 6 highly up‐regulated genes had no annotation. (**B**) The 60 most down‐regulated genes in shoots infested by *S. frugiperda* , included 15 genes involved in transcription regulation, 12 genes involved in defense response, and 5 genes involved in primary metabolism that were highly suppressed by *S. frugiperda* feeding. They comprised a group of MYB‐related transcription factors (Zm00001d026017, Zm00001d002545, and Zm00001d037998) and genes involved in the biosynthesis of DIBOA‐glucoside (cytochrome P450 71A26, Zm00001d035178), auxin (monooxygenase, Zm00001d021444) and starch (α‐amylase 3 chloroplastic, Zm00001d043662). A total of 19 highly down‐regulated genes had no annotation.
**Figure S4** KEGG pathway enrichment analysis of differentially expressed genes (DEGs) in maize induced by *D. v. virgifera* herbivory. (**A**) The top 20 enriched KEGG pathways in maize roots between *D. v. virgifera* herbivory (DV) and non‐manipulated control (C). (**B**) The top 20 enriched KEGG pathways in maize roots between *D. v. virgifera* herbivory and artificial root damage (MR). (**C**) The enriched KEGG pathways in shoot of maize seedling between *D. v. virgifera* herbivory and control. Enrichment scores are shown as ‐log10(adjusted *P* value). Number of DEGs involved in each pathway are shown above the bar.
**Figure S5** Heatmap of the relative expression levels (fold change after log2 transformation) of the 60 most up‐ (**A**) and down‐regulated (**B**) genes in maize roots induced by *Diabrotica virgifera virfigera* infestation relative to control. Color bar after gene ID and pie chart under heatmap showing the potential gene function. (**A**) The top 60 up‐regulated genes in roots induced by *D. v. virgifera* infestation included 21 defense‐related genes involved in the biosynthesis of dhurrin (*CYP79A33*, Zm00001d013753), suberin (O‐methyltransferase *ZRP4*, Zm00001d038703), DIBOA‐glucoside (tryptophan synthase, Zm00001d034461 and Zm00001d034453), isoflavonoid (Zm00001d016151), ethylene (1‐aminocyclopropane‐1‐carboxylate oxidase3, Zm00001d024852), auxin (tyrosine decarboxylase 1, Zm00001d024665), benzoxazinone (benzoxazinone synthesis14, *BX14*, Zm00001d004921), proteinase inhibitors (Zm00001d048656, 95 Zm00001d008548, Zm00001d048660) and so on, and 13 volatile emission‐related genes (six terpene synthase genes, one β‐caryophyllene synthase gene, two salicylate methyltransferase genes, one linalool synthase gene, *CYP92C5*, one NAD(P)‐binding Rossmann‐fold superfamily protein related to (Z)‐3‐hexen‐1‐yl acetate production, and one HXXXD‐type acyl‐transferase related to volatile benzenoid biosynthesis). Of these direct and indirect defense‐related genes in roots, polyphenol oxidase1 (Zm00001d000001), 17.4 kDa class I heat shock protein (Zm00001d028561), NAD(P)‐binding Rossmann‐fold superfamily protein (Zm00001d006796) and salicylate methyltransferase (Zm00001d052828) were induced by both shoot and root herbivory. Notably, a number of growth and development‐related genes such as GDSL esterase/lipase (Zm00001d023984), oil body‐associated protein (Zm00001d051459) and several genes coding for embryo protein (Zm00001d025434, Zm00001d037985, Zm00001d043709, Zm00001d002360) and seed maturation protein (Zm00001d026037, Zm00001d024414) were also induced in roots by above‐ and belowground herbivory. (**B**) From the 60 most down‐regulated genes in roots in response to *D. v. virgifera* infestation, many of them were only significantly suppressed by *D. v. virgifera* herbivory, whereas the expression of two potential defense‐related genes (Leucine‐rich repeat [LRR] family protein, Zm00001d026617; cysteine proteinase inhibitor, Zm00001d014219), one pumilio homolog 3 (Zm00001d018925) and three genes without annotation (Zm00001d046137, Zm00001d007955, Zm00001d045022) were significantly suppressed by both below‐ and aboveground herbivory. A group of genes involved in photosynthesis (such as chlorophyll a‐b binding protein, Zm00001d009589; photosystem I subunit O, Zm00001d003767) and metabolism of prophyrin and chlorophyll (protochlorophyllide reductase1, Zm00001d001820) showed extremely low expression levels in roots compared to their expression in leaves. The expression of these genes in maize roots was down‐regulated in response to *D. v. virgifera* infestation.
**Table S2** Primers used for qRT‐PCR.

**Table S1** Summary of RNA sequencing and mapping using the maize genome as the reference. The numerical values 1, 2, 3, 4 indicate the different biological replicates.

## Data Availability

The raw transcriptome data were deposited in the NCBI short read archive (SRA) with accession number PRJNA675077.

## References

[pld3426-bib-0001] Acosta, I. F. , Gasperini, D. , Chételat, A. , Stolz, S. , Santuari, L. , & Farmer, E. E. (2013). Role of NINJA in root jasmonate signaling. Proceedings of the National Academy of Sciences of the United States of America, 110, 15473–15478. 10.1073/pnas.1307910110 24003128 PMC3780868

[pld3426-bib-0002] Alborn, H. T. , Turlings, T. C. J. , Jones, T. H. , Stenhagen, G. , Loughrin, J. H. , & Tumlinson, J. H. (1997). An elicitor of plant volatiles from beet armyworm Oral secretion. Science, 276(5314), 945–949. 10.1126/science.276.5314.945

[pld3426-bib-0003] Anders, S. , Pyl, P. T. , & Huber, W. (2015). HTSeq‐A Python framework to work with high‐throughput sequencing data. Bioinformatics, 31, 166–169. 10.1093/bioinformatics/btu638 25260700 PMC4287950

[pld3426-bib-0004] Benjamini, Y. , & Hochberg, Y. (1995). Controlling the false discovery rate: A practical and powerful approach to multiple testing. Journal of the Royal Statistical Society, Series B, 57, 289–300.

[pld3426-bib-0005] Bernklau, E. J. , Hibbard, B. E. , Norton, A. P. , & Bjostad, L. B. (2016). Methyl anthranilate as a repellent for Western corn rootworm larvae (Coleoptera: Chrysomelidae). Journal of Economic Entomology, 109(4), 1683–1690. 10.1093/jee/tow090 27122493

[pld3426-bib-0006] Bianchet, C. , Wong, A. , Quaglia, M. , Alqurashi, M. , Gehring, C. , Ntoukakis, V. , & Pasqualini, S. (2019). An *Arabidopsis thaliana* leucine‐rich repeat protein harbors an adenylyl cyclase catalytic center and affects responses to pathogens. Journal of Plant Physiology, 232, 12–22. 10.1016/j.jplph.2018.10.025 30530199

[pld3426-bib-0007] Block, A. K. , Vaughan, M. M. , Schmelz, E. A. , & Christensen, S. A. (2019). Biosynthesis and function of terpenoid defense compounds in maize (*Zea mays*). Planta, 249(1), 21–30. 10.1007/s00425-018-2999-2 30187155

[pld3426-bib-0008] Bodenhausen, N. , & Reymond, P. (2007). Signaling pathways controlling induced resistance to insect herbivores in *Arabidopsis* . Molecular Plant‐Microbe Interactions, 20(11), 1406–1420. 10.1094/MPMI-20-11-1406 17977152

[pld3426-bib-0009] Bozorov, T. A. , Dinh, S. T. , & Baldwin, I. T. (2017). JA but not JA‐Ile is the cell‐nonautonomous signal activating JA mediated systemic defenses to herbivory in *Nicotiana attenuata* . Journal of Integrative Plant Biology, 59(8), 552–571. 10.1111/jipb.12545 28422432

[pld3426-bib-0010] Broekgaarden, C. , Caarls, L. , Vos, I. A. , Pieterse, C. M. J. , & Van Wees, S. C. M. (2015). Ethylene: Traffic controller on hormonal crossroads to defense. Plant Physiology, 169, 2371–2379. 10.1104/pp.15.01020 26482888 PMC4677896

[pld3426-bib-0011] Castellano, M. D. M. , Boniotti, M. B. , Caro, E. , Schnittger, A. , & Gutierrez, C. (2004). DNA replication licensing affects cell proliferation or endoreplication in a cell type‐specific manner. Plant Cell, 16(9), 2380–2393. 10.1105/tpc.104.022400 15316110 PMC520940

[pld3426-bib-0012] Christensen, S. A. , Huffaker, A. , Kaplan, F. , Sims, J. , Ziemann, S. , Doehlemann, G. , Ji, L. , Schmitz, R. J. , Kolomiets, M. V. , Alborn, H. T. , Mori, N. , Jander, G. , Ni, X. , Sartor, R. C. , Byers, S. , Abdo, Z. , & Schmelz, E. A. (2015). Maize death acids, 9‐lipoxygenase‐derived cyclopente(a)nones, display activity as cytotoxic phytoalexins and transcriptional mediators. Proceedings of the National Academy of Sciences of the United States of America, 112, 11407–11412. 10.1073/pnas.1511131112 26305953 PMC4568653

[pld3426-bib-0013] Christensen, S. A. , Nemchenko, A. , Borrego, E. , Murray, I. , Sobhy, I. S. , Bosak, L. , Deblasio, S. , Erb, M. , Robert, C. A. M. , Vaughn, K. A. , DeBlasio, S. , Herrfurth, C. , Tumlinson, J. , Feussner, I. , Jackson, D. , Turlings, T. C. J. , Engelberth, J. , Nansen, C. , Meeley, R. , & Kolomiets, M. V. (2013). The maize lipoxygenase, *ZmLOX10*, mediates green leaf volatile, jasmonate and herbivore‐induced plant volatile production for defense against insect attack. The Plant Journal, 74(1), 59–73. 10.1111/tpj.12101 23279660

[pld3426-bib-0014] Chuang, W. P. , Herde, M. , Ray, S. , Castano‐Duque, L. , Howe, G. A. , & Luthe, D. S. (2014). Caterpillar attack triggers accumulation of the toxic maize protein RIP2. The New Phytologist, 201(3), 928–939. 10.1111/nph.12581 24304477

[pld3426-bib-0015] De Lange, E. S. , Laplanche, D. , Guo, H. , Xu, W. , Vlimant, M. , Erb, M. , Ton, J. , & Turlings, T. C. J. (2020). *Spodoptera frugiperda* caterpillars suppress herbivore‐induced volatile emissions in maize. Journal of Chemical Ecology, 46, 344–360. 10.1007/s10886-020-01153-x 32002720

[pld3426-bib-0016] Dempsey, D. A. , Vlot, A. C. , Wildermuth, M. C. , & Klessig, D. F. (2011). Salicylic acid biosynthesis and metabolism. Arabidopsis Book, 9, e0156. 10.1199/tab.0156 22303280 PMC3268552

[pld3426-bib-0017] Dicke, M. , & Baldwin, I. T. (2010). The evolutionary context for herbivore‐induced plant volatiles: Beyond the ‘cry for help’. Trends in Plant Science, 15(3), 167–175. 10.1016/j.tplants.2009.12.002 20047849

[pld3426-bib-0018] Dicke, M. , & Sabelis, M. W. (1988). How plants obtain predatory mites as a bodyguard. Netherlands Journal of Zoology, 38, 148–165.

[pld3426-bib-0019] Engelberth, J. , Alborn, H. T. , Schmelz, E. A. , & Tumlinson, J. H. (2004). Airborne signals prime plants against insect herbivore attack. Proceedings of the National Academy of Sciences of the United States of America, 101, 1781–1785. 10.1073/pnas.0308037100 14749516 PMC341853

[pld3426-bib-0020] Erb, M. , Flors, V. , Karlen, D. , De Lange, E. , Planchamp, C. , D'Alessandro, M. , Turlings, T. C. J. , & Ton, J. (2009). Signal signature of aboveground‐induced resistance upon belowground herbivory in maize. The Plant Journal, 59, 292–302. 10.1111/j.1365-313X.2009.03868.x 19392694

[pld3426-bib-0021] Erb, M. , Glauser, G. , & Robert, C. A. M. (2012). Induced immunity against belowground insect herbivores—Activation of defenses in the absence of a jasmonate burst. Journal of Chemical Ecology, 38, 629–640. 10.1007/s10886-012-0107-9 22527052

[pld3426-bib-0022] Erb, M. , Gordon‐Weeks, R. , Flors, V. , Camañes, G. , Turlings, T. C. J. , & Ton, J. (2009). Belowground ABA boosts aboveground production of DIMBOA and primes induction of chlorogenic acid in maize. Plant Signaling & Behavior, 4(7), 636–638. 10.4161/psb.4.7.8973 19820311 PMC2710561

[pld3426-bib-0023] Erb, M. , Köllner, T. G. , Degenhardt, J. , Zwahlen, C. , Hibbard, B. E. , & Turlings, T. C. J. (2011). The role of abscisic acid and water stress in root herbivore‐induced leaf resistance. The New Phytologist, 189, 308–320. 10.1111/j.1469-8137.2010.03450.x 20840610

[pld3426-bib-0024] Erb, M. , Meldau, S. , & Howe, G. A. (2012). Role of phytohormones in insect‐specific plant reactions. Trends in Plant Science, 17(5), 250–259. 10.1016/j.tplants.2012.01.003 22305233 PMC3346861

[pld3426-bib-0025] Erb, M. , & Reymond, P. (2019). Molecular interactions between plants and insect herbivores. Annual Review of Plant Biology, 70(1), 527–557. 10.1146/annurev-arplant-050718-095910 30786233

[pld3426-bib-0026] Erb, M. , Robert, C. A. M. , Hibbard, B. E. , & Turlings, T. C. J. (2011). Sequence of arrival determines plant‐mediated interactions between herbivores. Journal of Ecology, 99, 7–15. 10.1111/j.1365-2745.2010.01757.x

[pld3426-bib-0027] Erb, M. , Veyrat, N. , Robert, C. A. M. , Xu, H. , Frey, M. , Ton, J. , & Turlings, T. C. J. (2015). Indole is an essential herbivore‐induced volatile priming signal in maize. Nature Communications, 6, 6273. 10.1038/ncomms7273 PMC4339915 25683900

[pld3426-bib-0028] Frey, M. , Schullehner, K. , Dick, R. , Fiesselmann, A. , & Gierl, A. (2009). Benzoxazinoid biosynthesis, a model for evolution of secondary metabolic pathways in plants. Phytochemistry, 70(15–16), 1645–1651. 10.1016/j.phytochem.2009.05.012 19577780

[pld3426-bib-0029] Geng, P. , Zhang, S. , Liu, J. , Zhao, C. , Wu, J. , Cao, Y. , Fu, C. , Han, X. , He, H. , & Zhao, Q. (2020). MYB20, MYB42, MYB43, and MYB85 regulate phenylalanine and lignin biosynthesis during secondary cell wall formation. Plant Physiology, 182(3), 1272–1283. 10.1104/pp.19.01070 31871072 PMC7054866

[pld3426-bib-0030] Gil, K. E. , & Park, C. M. (2019). Thermal adaptation and plasticity of the plant circadian clock. The New Phytologist, 221(3), 1215–1229. 10.1111/nph.15518 30289568

[pld3426-bib-0031] Glauser, G. , Marti, G. , Villard, N. , Doyen, G. A. , Wolfender, J. L. , Turlings, T. C. J. , & Erb, M. (2011). Induction and detoxification of maize 1,4‐benzoxazin‐3‐ones by insect herbivores. The Plant Journal, 68(5), 901–911. 10.1111/j.1365-313X.2011.04740.x 21838747

[pld3426-bib-0032] Guo, J. , Qi, J. , He, K. , Wu, J. , Bai, S. , Zhang, T. , Zhao, J. , & Wang, Z. (2019). The Asian corn borer *Ostrinia furnacalis* feeding increases the direct and indirect defence of mid‐whorl stage commercial maize in the field. Plant Biotechnology Journal, 17, 88–102. 10.1111/pbi.12949 29754404 PMC6330542

[pld3426-bib-0033] Harfouche, A. L. , Shivaji, R. , Stocker, R. , Williams, P. W. , & Luthe, D. S. (2006). Ethylene signaling mediates a maize defense response to insect herbivory. Molecular Plant‐Microbe Interactions, 19, 189–199. 10.1094/MPMI-19-0189 16529381

[pld3426-bib-0034] Hasegawa, S. , Sogabe, Y. , Asano, T. , Nakagawa, T. , Nakamura, H. , Kodama, H. , Ohta, H. , Yamaguchi, K. , Mueller, M. J. , & Nishiuchi, T. (2011). Gene expression analysis of wounding‐induced root‐to‐shoot communication in *Arabidopsis thaliana* . Plant, Cell and Environment, 34(5), 705–716. 10.1111/j.1365-3040.2011.02274.x 21241326

[pld3426-bib-0035] Heil, M. (2015). Extrafloral nectar at the plant‐insect interface: A spotlight on chemical ecology, phenotypic plasticity, and food webs. Annual Review of Entomology, 60(1), 213–232. 10.1146/annurev-ento-010814-020753 25564741

[pld3426-bib-0036] Howe, G. A. , & Jander, G. (2008). Plant immunity to insect herbivores. Annual Review of Plant Biology, 59(1), 41–66. 10.1146/annurev.arplant.59.032607.092825 18031220

[pld3426-bib-0037] Huang, W. , Siemann, E. , Xiao, L. , Yang, X. , & Ding, J. (2014). Species‐specific defence responses facilitate conspecifics and inhibit heterospecifics in above–belowground herbivore interactions. Nature Communications, 5, 4851. 10.1038/ncomms5851 PMC4199110 25241651

[pld3426-bib-0038] Huang, W. , Wang, Y. , Li, X. , & Zhang, Y. (2020). Biosynthesis and regulation of salicylic acid and N‐hydroxypipecolic acid in plant immunity. Molecular Plant, 13, 31–41. 10.1016/j.molp.2019.12.008 31863850

[pld3426-bib-0039] Humphrey, P. T. , Gloss, A. D. , Alexandre, N. M. , Villalobos, M. M. , Fremgen, M. R. , Groen, S. C. , Meihls, L. N. , Jander, G. , & Whiteman, N. K. (2016). Aversion and attraction to harmful plant secondary compounds jointly shape the foraging ecology of a specialist herbivore. Ecology and Evolution, 6, 3256–3268. 10.1002/ece3.2082 27096082 PMC4829532

[pld3426-bib-0040] Jiao, Y. , Peluso, P. , Shi, J. , Liang, T. , Stitzer, M. C. , Wang, B. , Campbell, M. S. , Stein, J. C. , Wei, X. , Chin, C. S. , Guill, K. , Regulski, M. , Kumari, S. , Olson, A. , Gent, J. , Schneider, K. L. , Wolfgruber, T. K. , May, M. R. , Springer, N. M. , … Ware, D. (2017). Improved maize reference genome with single‐molecule technologies. Nature, 546(7659), 524–527. 10.1038/nature22971 28605751 PMC7052699

[pld3426-bib-0041] Johnson, S. N. , Erb, M. , & Hartley, S. E. (2016). Roots under attack: Contrasting plant responses to below‐ and aboveground insect herbivory. The New Phytologist, 210, 413–418. 10.1111/nph.13807 26781566

[pld3426-bib-0042] Kang, J. , Yu, H. , Tian, C. , Zhou, W. , Li, C. , Jiao, Y. , & Liu, D. (2014). Suppression of photosynthetic gene expression in roots is required for sustained root growth under phosphate deficiency. Plant Physiology, 165(3), 1156–1170. 10.1104/pp.114.238725 24868033 PMC4081329

[pld3426-bib-0043] Karssemeijer, P. N. , Reichelt, M. , Gershenzon, J. , van Loon, J. , & Dicke, M. (2020). Foliar herbivory by caterpillars and aphids differentially affects phytohormonal signalling in roots and plant defence to a root herbivore. Plant, Cell & Environment, 43(3), 775–786. 10.1111/pce.13707 PMC7065167 31873957

[pld3426-bib-0044] Kim, D. , Langmead, B. , & Salzberg, S. L. (2015). HISAT: A fast spliced aligner with low memory requirements. Nature Methods, 12, 357–360. 10.1038/nmeth.3317 25751142 PMC4655817

[pld3426-bib-0045] Köhler, A. , Maag, D. , Veyrat, N. , Glauser, G. , Wolfender, J. L. , Turlings, T. C. J. , & Erb, M. (2015). Within‐plant distribution of 1,4‐benzoxazin‐3‐ones contributes to herbivore niche differentiation in maize. Plant, Cell and Environment, 38(6), 1081–1093. 10.1111/pce.12464 25293400

[pld3426-bib-0046] Köllner, T. G. , Held, M. , Lenk, C. , Hiltpold, I. , Turlings, T. C. J. , Gershenzon, J. , & Degenhardt, J. (2008). A maize (E)‐β‐caryophyllene synthase implicated in indirect defense responses against herbivores is not expressed in most American maize varieties. Plant Cell, 20, 482–494. 10.1105/tpc.107.051672 18296628 PMC2276456

[pld3426-bib-0047] Köllner, T. G. , Lenk, C. , Zhao, N. , Seidl‐Adams, I. , Gershenzon, J. , Chen, F. , & Degenhardt, J. (2010). Herbivore‐induced SABATH methyltransferases of maize that methylate anthranilic acid using S‐adenosyl‐L‐methionine. Plant Physiology, 153(4), 1795–1807. 10.1104/pp.110.158360 20519632 PMC2923889

[pld3426-bib-0048] Koo, A. J. K. , & Howe, G. A. (2012). Catabolism and deactivation of the lipid‐derived hormone jasmonoyl‐isoleucine. Frontiers in Plant Science, 3, 1–7. 22639640 10.3389/fpls.2012.00019 PMC3355578

[pld3426-bib-0049] Kos, M. , Kabouw, P. , Noordam, R. , Hendriks, K. , Vet, L. E. M. , Van loon, J. J. A. , & Dicke, M. (2011). Prey‐mediated effects of glucosinolates on aphid predators. Ecological Entomology, 36, 377–388. 10.1111/j.1365-2311.2011.01282.x

[pld3426-bib-0050] Kumar, P. , Pandit, S. S. , Steppuhn, A. , & Baldwin, I. T. (2014). Natural history‐driven, plant‐mediated RNAi‐based study reveals *CYP6B46*'s role in a nicotine‐mediated antipredator herbivore defense. Proceedings of the National Academy of Sciences of the United States of America, 111, 1245–1252. 10.1073/pnas.1314848111 24379363 PMC3910579

[pld3426-bib-0051] Leng, P. , Yuan, B. , Guo, Y. , & Chen, P. (2014). The role of abscisic acid in fruit ripening and responses to abiotic stress. Journal of Experimental Botany, 65, 4577–4588. 10.1093/jxb/eru204 24821949

[pld3426-bib-0052] Li, B. , Fan, R. , Guo, S. , Wang, P. , Zhu, X. , Fan, Y. , Chen, Y. , He, K. , Kumar, A. , Shi, J. , Wang, Y. , Li, L. , Hu, Z. , & Song, C. P. (2019). The Arabidopsis MYB transcription factor, MYB111 modulates salt responses by regulating flavonoid biosynthesis. Environmental and Experimental Botany, 166, 103807. 10.1016/j.envexpbot.2019.103807

[pld3426-bib-0053] Lortzing, T. , & Steppuhn, A. (2016). Jasmonate signalling in plants shapes plant‐insect interaction ecology. Current Opinion in Insect Science, 14, 32–39. 10.1016/j.cois.2016.01.002 27436644

[pld3426-bib-0054] Love, M. I. , Huber, W. , & Anders, S. (2014). Moderated estimation of fold change and dispersion for RNA‐seq data with DESeq2. Genome Biology, 15(12), 1–21. 10.1186/s13059-014-0550-8 PMC4302049 25516281

[pld3426-bib-0055] Lu, J. , Li, J. , Ju, H. , Liu, X. , Erb, M. , Wang, X. , & Lou, Y. (2014). Contrasting effects of ethylene biosynthesis on induced plant resistance against a chewing and a piercing‐sucking herbivore in rice. Molecular Plant, 7, 1670–1682. 10.1093/mp/ssu085 25064847

[pld3426-bib-0056] Lu, J. , Robert, C. A. M. , Riemann, M. , Cosme, M. , Mène‐Saffrané, L. , Massana, J. , Stout, M. J. , Lou, Y. , Gershenzon, J. , & Erb, M. (2015). Induced jasmonate signaling leads to contrasting effects on root damage and herbivore performance. Plant Physiology, 167(3), 1100–1116. 10.1104/pp.114.252700 25627217 PMC4348761

[pld3426-bib-0057] Maag, D. , Köhler, A. , Robert, C. A. M. , Frey, M. , Wolfender, J. L. , Turlings, T. C. J. , Glauser, G. , & Erb, M. (2016). Highly localized and persistent induction of Bx1‐dependent herbivore resistance factors in maize. The Plant Journal, 88(6), 976–991. 10.1111/tpj.13308 27538820

[pld3426-bib-0058] Machado, R. A. R. , Arce, C. C. M. , McClure, M. A. , Baldwin, I. T. , & Erb, M. (2018). Aboveground herbivory induced jasmonates disproportionately reduce plant reproductive potential by facilitating root nematode infestation. Plant, Cell & Environment, 41, 797–808. 10.1111/pce.13143 29327360

[pld3426-bib-0059] Machado, R. A. R. , Theepan, V. , Robert, C. A. M. , Züst, T. , Hu, L. , Su, Q. , Schimmel, B. C. J. , & Erb, M. (2021). The plant metabolome guides fitness‐relevant foraging decisions of a specialist herbivore. PLoS Biology, 19, e3001114. 10.1371/journal.pbio.3001114 33600420 PMC7924754

[pld3426-bib-0060] Miles, C. I. , del Campo, M. L. , & Renwick, J. A. A. (2005). Behavioral and chemosensory responses to a host recognition cue by larvae of *Pieris rapae* . Journal of Comparative Physiology A, 191, 147–155. 10.1007/s00359-004-0580-x 15711970

[pld3426-bib-0061] Mithöfer, A. , & Boland, W. (2012). Plant defense against herbivores: Chemical aspects. Annual Review of Plant Biology, 63(1), 431–450. 10.1146/annurev-arplant-042110-103854 22404468

[pld3426-bib-0062] Mutti, N. S. , Louis, J. , Pappan, L. K. , Pappan, K. , Begum, K. , Chen, M. S. , Park, Y. , Dittmer, N. , Marshall, J. , Reese, J. C. , & Reeck, G. R. (2008). A protein from the salivary glands of the pea aphid, *Acyrthosiphon pisum*, is essential in feeding on a host plant. Proceedings of the National Academy of Sciences of the United States of America, 105, 9965–9969. 10.1073/pnas.0708958105 18621720 PMC2481341

[pld3426-bib-0063] Olds, C. L. , Glennon, E. K. K. , & Luckhart, S. (2018). Abscisic acid: New perspectives on an ancient universal stress signaling molecule. Microbes and Infection, 20(9–10), 484–492. 10.1016/j.micinf.2018.01.009 29408537

[pld3426-bib-0064] Pan, Y. , Zhao, S. , Wang, Z. , Wang, X. , Zhang, X. , Lee, Y. , & Xi, J. (2020). Quantitative proteomics suggests changes in the carbohydrate metabolism of maize in response to larvae of the belowground herbivore *Holotrichia parallela* . PeerJ, 8, 1–14. 10.7717/peerj.9819 PMC7456535 32913681

[pld3426-bib-0065] Pechan, T. , Cohen, A. , Williams, W. P. , & Luthe, D. S. (2002). Insect feeding mobilizes a unique plant defense protease that disrupts the peritrophic matrix of caterpillars. Proceedings of the National Academy of Sciences of the United States of America, 99, 13319–13323. 10.1073/pnas.202224899 12235370 PMC130631

[pld3426-bib-0066] Qi, J. , Sun, G. , Wang, L. , Zhao, C. , Hettenhausen, C. , Schuman, M. C. , Baldwin, I. T. , Li, J. , Song, J. , Liu, Z. , Xu, G. , Lu, X. , & Wu, J. (2016). Oral secretions from *Mythimna separata* insects specifically induce defence responses in maize as revealed by high‐dimensional biological data. Plant, Cell & Environment, 39(8), 1749–1766. 10.1111/pce.12735 PMC5295635 26991784

[pld3426-bib-0067] Rasmann, S. , Köllner, T. G. , Degenhardt, J. , Hiltpold, I. , Toepfer, S. , Kuhlmann, U. , Gershenzon, J. , & Turlings, T. C. J. (2005). Recruitment of entomopathogenic nematodes by insect‐damaged maize roots. Nature, 434(7034), 732–737. 10.1038/nature03451 15815622

[pld3426-bib-0068] Rasmann, S. , & Turlings, T. C. J. (2008). First insights into specificity of belowground tritrophic interactions. Oikos, 117, 362–369. 10.1111/j.2007.0030-1299.16204.x

[pld3426-bib-0069] Renwick, J. A. A. , & Lopez, K. (1999). Experience‐based food consumption by larvae of *Pieris rapae*: Addiction to glucosinolates? Entomologia Experimentalis et Applicata, 91, 51–58. 10.1007/978-94-017-1890-5_6

[pld3426-bib-0070] Richter, A. , Schaff, C. , Zhang, Z. , Lipka, A. E. , Tian, F. , Köllner, T. G. , Schnee, C. , Preiß, S. , Irmisch, S. , Jander, G. , Boland, W. , Gershenzon, J. , Buckler, E. S. , & Degenhardt, J. (2016). Characterization of biosynthetic pathways for the production of the volatile homoterpenes DMNT and TMTT in *Zea mays* . Plant Cell, 28(10), 2651–2665. 10.1105/tpc.15.00919 27662898 PMC5134970

[pld3426-bib-0071] Robert, C. A. M. , Ferrieri, R. A. , Schirmer, S. , Babst, B. A. , Schueller, M. J. , Machado, R. A. R. , Arce, C. C. M. , Hibbard, B. E. , Gershenzon, J. , Turlings, T. C. J. , Robert, C. A. M. , Ferrieri, R. A. , Schirmer, S. , Babst, B. A. , Schueller, M. J. , Machado, R. A. R. , Arce, C. C. M. , Hibbard, B. E. , Gershenzon, J. , … Erb, M. (2014). Induced carbon reallocation and compensatory growth as root herbivore tolerance mechanisms. Plant, Cell & Environment, 37(11), 2613–2622. 10.1111/pce.12359 24762051

[pld3426-bib-0072] Robert, C. A. M. , Veyrat, N. , Glauser, G. , Marti, G. , Doyen, G. R. , Villard, N. , Gaillard, M. D. P. , Köllner, T. G. , Giron, D. , Body, M. , Babst, B. A. , Ferrieri, R. A. , Turlings, T. C. J. , & Erb, M. (2012). A specialist root herbivore exploits defensive metabolites to locate nutritious tissues. Ecology Letters, 15, 55–64. 10.1111/j.1461-0248.2011.01708.x 22070646

[pld3426-bib-0073] Schmelz, E. A. , Alborn, H. T. , Banchio, E. , & Tumlinson, J. H. (2003). Quantitative relationships between induced jasmonic acid levels and volatile emission in *Zea mays* during *Spodoptera exigua* herbivory. Planta, 216(4), 665–673. 10.1007/s00425-002-0898-y 12569409

[pld3426-bib-0074] Schmelz, E. A. , Carroll, M. J. , LeClere, S. , Phipps, S. M. , Meredith, J. , Chourey, P. S. , Alborn, H. T. , & Teal, P. E. A. (2006). Fragments of ATP synthase mediate plant perception of insect attack. Proceedings of the National Academy of Sciences of the United States of America, 103, 8894–8899. 10.1073/pnas.0602328103 16720701 PMC1482674

[pld3426-bib-0075] Schmelz, E. A. , Engelberth, J. , Alborn, H. T. , & Teal, P. E. A. (2009). Phytohormone‐based activity mapping of insect herbivore‐produced elicitors. Proceedings of the National Academy of Sciences of the United States of America, 106, 653–657. 10.1073/pnas.0811861106 19124770 PMC2626758

[pld3426-bib-0076] Schnee, C. , Köllner, T. G. , Held, M. , Turlings, T. C. J. , Gershenzon, J. , & Degenhardt, J. (2006). The products of a single maize sesquiterpene synthase form a volatile defense signal that attracts natural enemies of maize herbivores. Proceedings of the National Academy of Sciences of the United States of America, 103, 1129–1134. 10.1073/pnas.0508027103 16418295 PMC1347987

[pld3426-bib-0077] Schuman, M. C. , & Baldwin, I. T. (2016). The layers of plant responses to insect herbivores. Annual Review of Entomology, 61(1), 373–394. 10.1146/annurev-ento-010715-023851 26651543

[pld3426-bib-0078] Singer, M. S. , Mace, K. C. , & Bernays, E. A. (2009). Self‐medication as adaptive plasticity: Increased ingestion of plant toxins by parasitized caterpillars. PLoS ONE, 4(3), e4796. 10.1371/journal.pone.0004796 19274098 PMC2652102

[pld3426-bib-0079] Smilanich, A. M. , Dyer, L. A. , Chambers, J. Q. , & Bowers, M. D. (2009). Immunological cost of chemical defence and the evolution of herbivore diet breadth. Ecology Letters, 12, 612–621. 10.1111/j.1461-0248.2009.01309.x 19392713

[pld3426-bib-0080] Song, S. , Huang, H. , Gao, H. , Wang, J. , Wu, D. , Liu, X. , Yang, S. , Zhai, Q. , Li, C. , Qi, T. , & Xie, D. (2014). Interaction between MYC2 and ETHYLENE INSENSITIVE3 modulates antagonism between jasmonate and ethylene signaling in *Arabidopsis* . Plant Cell, 26(1), 263–279. 10.1105/tpc.113.120394 24399301 PMC3963574

[pld3426-bib-0081] Sternberg, E. D. , Lefèvre, T. , Li, J. , de Castillejo, C. L. F. , Li, H. , Hunter, M. D. , & de Roode, J. C. (2012). Food plant derived disease tolerance and resistance in a natural butterfly‐plant‐parasite interactions. Evolution, 66(11), 3367–3376. 10.1111/j.1558-5646.2012.01693.x 23106703

[pld3426-bib-0082] Stotz, H. U. , Pittendrigh, B. R. , Kroymann, J. , Weniger, K. , Fritsche, J. , Bauke, A. , & Mitchell‐Olds, T. (2000). Induced plant defense responses against chewing insects. Ethylene signaling reduces resistance of Arabidopsis against Egyptian cotton worm but not diamondback moth. Plant Physiology, 124(3), 1007–1018. 10.1104/pp.124.3.1007 11080278 PMC59200

[pld3426-bib-0083] Stracke, R. , Jahns, O. , Keck, M. , Tohge, T. , Niehaus, K. , Fernie, A. R. , & Weisshaar, B. (2010). Analysis of production of flavonol glycosides‐dependent flavonol glycoside accumulation in *Arabidopsis thaliana* plants reveals MYB11‐, MYB12‐ and MYB111‐independent flavonol glycoside accumulation. The New Phytologist, 188(4), 985–1000. 10.1111/j.1469-8137.2010.03421.x 20731781

[pld3426-bib-0084] Tamaoki, M. (2008). The role of phytohormone signaling in ozone‐induced cell death in plants. Plant Signaling & Behavior, 3, 166–174. 10.4161/psb.3.3.5538 19513211 PMC2634110

[pld3426-bib-0085] Tamayo, M. C. , Rufat, M. , Bravo, J. M. , & San Segundo, B. (2000). Accumulation of a maize proteinase inhibitor in response to wounding and insect feeding, and characterization of its activity toward digestive proteinases of *Spodoptera littoralis* larvae. Planta, 211(1), 62–71. 10.1007/s004250000258 10923704

[pld3426-bib-0086] Tamiru, A. , Bruce, T. J. A. , Woodcock, C. M. , Caulfield, J. C. , Midega, C. A. O. , Ogol, C. K. P. O. , Mayon, P. , Birkett, M. A. , Pickett, J. A. , & Khan, Z. R. (2011). Maize landraces recruit egg and larval parasitoids in response to egg deposition by a herbivore. Ecology Letters, 14, 1075–1083. 10.1111/j.1461-0248.2011.01674.x 21831133

[pld3426-bib-0087] Tao, L. , & Hunter, M. D. (2013). Allocation of resources away from sites of herbivory under simultaneous attack by aboveground and belowground herbivores in the common milkweed, *Asclepias syriaca* . Arthropod‐Plant Interactions, 7, 217–224. 10.1007/s11829-012-9235-y

[pld3426-bib-0088] Ton, J. , DAlessandro, M. , Jourdie, V. , Jakab, G. , Karlen, D. , Held, M. , Mauch‐Mani, B. , & Turlings, T. C. J. (2007). Priming by airborne signals boosts direct and indirect resistance in maize. The Plant Journal, 49, 16–26. 10.1111/j.1365-313X.2006.02935.x 17144894

[pld3426-bib-0089] Tretner, C. , Huth, U. , & Hause, B. (2008). Mechanostimulation of *Medicago truncatula* leads to enhanced levels of jasmonic acid. Journal of Experimental Botany, 59, 2847–2856. 10.1093/jxb/ern145 18540020 PMC2486479

[pld3426-bib-0090] Turlings, T. C. J. , Alborn, H. T. , Loughrin, J. H. , & Tumlinson, J. H. (2000). Volicitin, an elicitor of maize volatiles in Oral secretion of *Spodoptera Exigua*: Isolation and bioactivity. Journal of Chemical Ecology, 26, 189–202. 10.1023/A:1005449730052

[pld3426-bib-0091] Turlings, T. C. J. , Davison, A. C. , & Tamò, C. (2004). A six‐arm olfactometer permitting simultaneous observation of insect attraction and odour trapping. Physiological Entomology, 29, 45–55. 10.1111/j.1365-3032.2004.0362.x

[pld3426-bib-0092] Turlings, T. C. J. , & Erb, M. (2018). Tritrophic interactions mediated by herbivore‐induced plant volatiles: Mechanisms, ecological relevance, and application potential. Annual Review of Entomology, 63(1), 433–452. 10.1146/annurev-ento-020117-043507 29324043

[pld3426-bib-0093] Turlings, T. C. J. , Tumlinson, J. H. , & Lewis, W. J. (1990). Exploitation of herbivore‐induced plant odors by host‐seeking parasitic wasps. Science, 250(4985), 1251–1253. 10.1126/science.250.4985.1251 17829213

[pld3426-bib-0094] Tzin, V. , Fernandez‐Pozo, N. , Richter, A. , Schmelz, E. A. , Schoettner, M. , Schäfer, M. , Ahern, K. R. , Meihls, L. N. , Kaur, H. , Huffaker, A. , Mori, N. , Degenhardt, J. , Mueller, L. A. , & Jander, G. (2015). Dynamic maize responses to aphid feeding are revealed by a time series of transcriptomic and metabolomic assays. Plant Physiology, 169, 1727–1743. 10.1104/pp.15.01039 26378100 PMC4634079

[pld3426-bib-0095] Tzin, V. , Hojo, Y. , Strickler, S. R. , Bartsch, L. J. , Archer, C. M. , Ahern, K. R. , Zhou, S. , Christensen, S. A. , Galis, I. , Mueller, L. A. , & Jander, G. (2017). Rapid defense responses in maize leaves induced by *Spodoptera exigua* caterpillar feeding. Journal of Experimental Botany, 68, 4709–4723. 10.1093/jxb/erx274 28981781 PMC5853842

[pld3426-bib-0096] Visakorpi, K. , Gripenberg, S. , Malhi, Y. , Bolas, C. , Oliveras, I. , Harris, N. , Rifai, S. , & Riutta, T. (2018). Small‐scale indirect plant responses to insect herbivory could have major impacts on canopy photosynthesis and isoprene emission. The New Phytologist, 220(3), 799–810. 10.1111/nph.15338 30047151

[pld3426-bib-0097] Vos, I. A. , Verhage, A. , Schuurink, R. C. , Watt, L. G. , Pieterse, C. M. J. , & Van Wees, S. C. M. (2013). Onset of herbivore‐induced resistance in systemic tissue primed for jasmonate‐dependent defenses is activated by abscisic acid. Frontiers in Plant Science, 4, 1–10. 24416038 10.3389/fpls.2013.00539 PMC3874679

[pld3426-bib-0098] Wang, K. , Guo, Q. , Froehlich, J. E. , Hersh, H. L. , Zienkiewicz, A. , Howe, G. A. , & Benning, C. (2018). Two abscisic acid‐responsive plastid lipase genes involved in jasmonic acid biosynthesis in *Arabidopsis thaliana* . Plant Cell, 30(5), 1006–1022. 10.1105/tpc.18.00250 29666162 PMC6002186

[pld3426-bib-0099] Woldemariam, M. G. , Ahern, K. , Jander, G. , & Tzin, V. (2018). A role for 9‐lipoxygenases in maize defense against insect herbivory. Plant Signaling & Behavior, 13, e1422462. 10.1080/15592324.2017.1422462 29293391 PMC5790410

[pld3426-bib-0100] Wu, J. , & Baldwin, I. T. (2010). New insights into plant responses to the attack from insect herbivores. Annual Review of Genetics, 44, 1–24. 10.1146/annurev-genet-102209-163500 20649414

[pld3426-bib-0101] Xiao, L. , Carrillo, J. , Siemann, E. , & Ding, J. (2019). Herbivore‐specific induction of indirect and direct defensive responses in leaves and roots. AoB PLANTS, 11, plz003. 10.1093/aobpla/plz003 30792834 PMC6378760

[pld3426-bib-0102] Xiao, Y. , Wang, Q. , Erb, M. , Turlings, T. C. J. , Ge, L. , Hu, L. , Li, J. , Han, X. , Zhang, T. , Lu, J. , Zhang, G. , & Lou, Y. (2012). Specific herbivore‐induced volatiles defend plants and determine insect community composition in the field. Ecology Letters, 15, 1130–1139. 10.1111/j.1461-0248.2012.01835.x 22804824

[pld3426-bib-0103] Xie, C. , Mao, X. , Huang, J. , Ding, Y. , Wu, J. , Dong, S. , Kong, L. , Gao, G. , Li, C. Y. , & Wei, L. (2011). KOBAS 2.0: A web server for annotation and identification of enriched pathways and diseases. Nucleic Acids Research, 39(suppl_2), 316–322. 10.1093/nar/gkr483 PMC3125809 21715386

[pld3426-bib-0104] Ye, M. , Kuai, P. , Hu, L. , Ye, M. , Sun, H. , Erb, M. , & Lou, Y. (2020). Suppression of a leucine‐rich repeat receptor‐like kinase enhances host plant resistance to a specialist herbivore. Plant, Cell & Environment, 43(10), 2571–2585. 10.1111/pce.13834 32598036

[pld3426-bib-0105] Ye, W. , Yu, H. , Jian, Y. , Zeng, J. , Ji, R. , Chen, H. , & Lou, Y. (2017). A salivary EF‐hand calcium‐binding protein of the brown planthopper *Nilaparvata lugens* functions as an effector for defense responses in rice. Scientific Reports, 7(1), 40498. 10.1038/srep40498 28098179 PMC5241783

[pld3426-bib-0106] Zheng, H. , Yang, Z. , Wang, W. , Guo, S. , Li, Z. , Liu, K. , & Sui, N. (2020). Transcriptome analysis of maize inbred lines differing in drought tolerance provides novel insights into the molecular mechanisms of drought responses in roots. Plant Physiology and Biochemistry, 149, 11–26. 10.1016/j.plaphy.2020.01.027 32035249

