# Supplementary material for: Belowground and aboveground herbivory differentially affect the transcriptome in roots and shoots of maize
Source: Plant Direct. 2022 Jul 22;6(7):e426. doi: 10.1002/pld3.426 (PMC9307387; doi:10.1002/pld3.426)
Supplement: Supplementary file 10 — Figure S1 Expression levels and correlations for maize genes from qRT‐PCR (left) and RNA‐seq gene expression data (right). The transcript levels (mean + SE, n = 4) of eight genes in shoots of maize seedlings are shown after 72 h of belowground infestation by Diabrotica virgifera virgifera (DV), aboveground infestation by Spodoptera frugiperda (SF), application of root (MR) or shoot mechanical damage (MS). Non‐treated seedlings served as controls (C). The following genes were measured: BX14 (benzoxazinone synthesis14, Zm00001d004921), ZRP4‐like (O‐methyltransferase, Zm00001d038703), PR5 (pathogenesis‐related protein5, Zm00001d031158), PR10 (pathogenesis related protein10, Zm00001d028816), LOX3 (lipoxygenase, Zm00001d033623), PPO (polyphenol oxidase, Zm00001d000001), BBTI13 (Bowman‐Birk type trypsin inhibitor, Zm00001d048660), CLH (chlorophyllase1, Zm00001d019758). For qRT‐PCR data, fold‐change of gene expression level was calculated using the 2−ΔΔCT method. The results (threshold cycle values) of the qRT‐PCR assays were normalized to the expression level of ZmCUL (cullin, Zm00001d024855). For RNA‐seq data, gene expression levels were calculated as FPKM (fragments per kilobase of transcript per million fragments mapped). Figure S2 KEGG pathway enrichment analysis of differentially expressed genes (DEGs) in maize induced by S. frugiperda herbivory. (A) The top 20 enriched KEGG pathways in maize shoot between S. frugiperda herbivory (SF) and non‐manipulated control (C). (B) The top 20 enriched KEGG pathways in maize shoot between S. frugiperda herbivory and artificial shoot damage (MS). (C) The enriched KEGG pathways in roots of maize seedlings between S. frugiperda herbivory and control. Enrichment scores are shown as ‐log10(adjusted P value). Number of DEGs involved in each pathway are shown above the bar. Figure S3 Heatmap of the relative expression levels (fold change after log2 transformation) of the 60 most up‐ (A) and down‐regulated (B) genes in maize shoot induc [file PLD3-6-e426-s004.pdf]

This PDF file includes:

Supplemental experimental procedures, Figures S1 to S5, and Table S2.

Table S1 and Data S1 to S9 are attached in separate documents.

Supplemental experimental procedures

Total RNA isolation and Real-Time qPCR

Frozen shoots and roots were ground into a fine powder in a mortar filled with liquid nitrogen. Total RNA was isolated using the GeneJET Plant Purification Mini Kit (Thermo Fisher Scientific Baltics UAB, Vilnius, Lithuania) according to the manufacturer's instructions and complete DNA removal was performed using the RNase-Free DNase Set (QIAGEN, Hilden, Germany). The potential RNA degradation and contamination were preliminarily checked with 1% agarose gel electrophoresis. Total RNA concentration and purity were determined using NanoDrop 2000 spectrophotometer (ThermoFisher Scientific Inc., Waltham, MA, USA) and the integrity of each RNA sample was assessed by using the RNA Nano 6000 Assay Kit of Agilent 2100 bioanalyzer (Agilent Technologies, Palo Alto, CA, USA). Each total RNA sample (500 ng) was reverse transcribed using the GoScript™ Reverse Transcription System (Promega). Real-time qPCR was performed on the Rotor-Gene™ 6000 (Corbett Research) using GoTaq® qPCR Master Mix (Promega). Primers used for real-time qPCR are listed in Table S2. For the expression analysis of each gene, samples from uninfested control maize plants were designated as calibrator. Relative expression levels of each gene were normalized with the ZmCUL gene (Cullin, locus tag: Zm00001d024855) (Manoli et al., 2012) and calculated using the $2^{-\triangle\triangle Ct}$ method (Livak and Schmittgen, 2001). Tissue from three individual maize seedlings was combined into one replicate, and four independent biological replicates per treatment were analyzed.

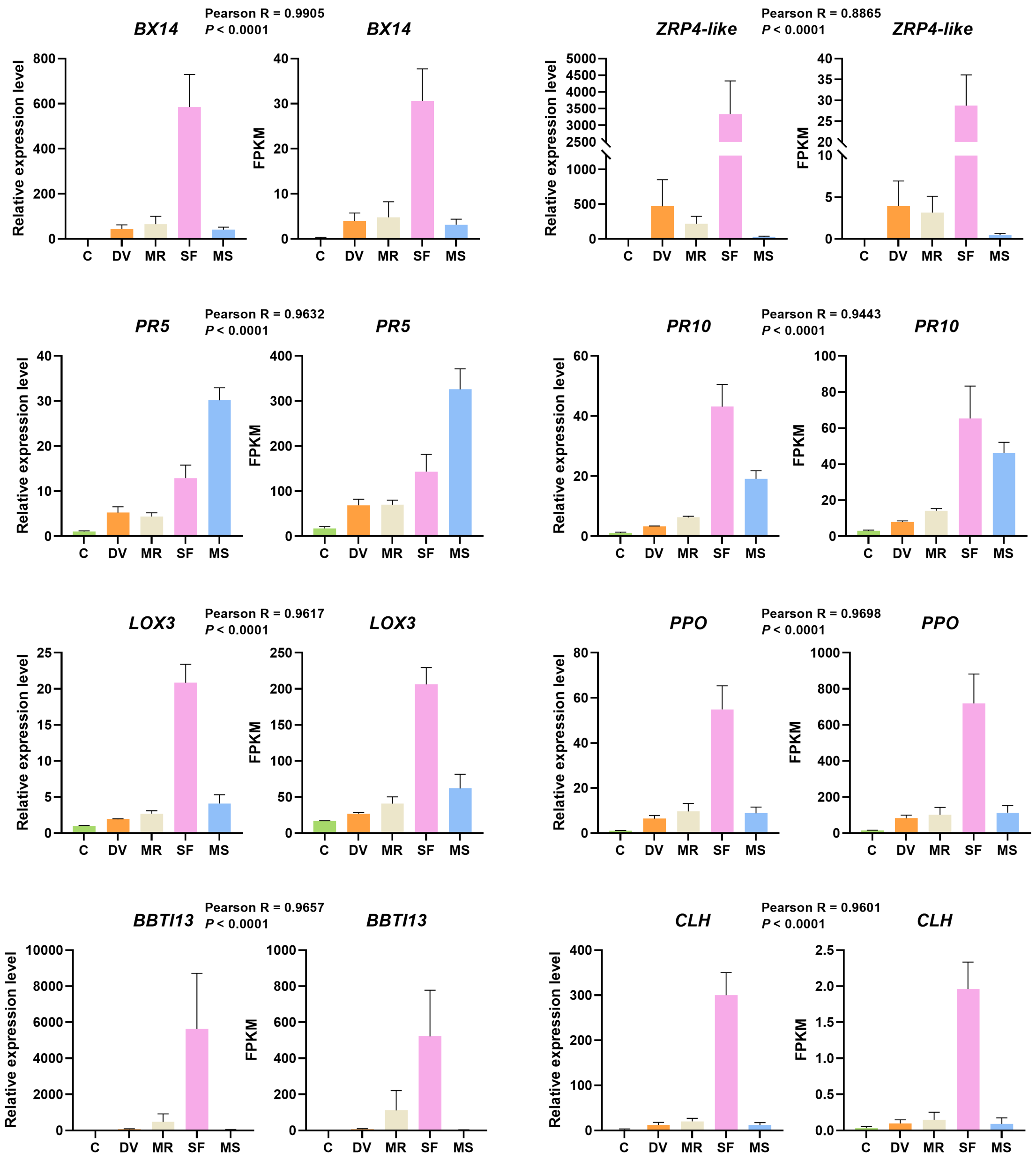

Figure S1 Expression levels and correlations for maize genes from qRT-PCR (left) and RNA-seq gene expression data (right). The transcript levels (mean + SE, *n* = 4) of eight genes in shoots of maize seedlings are shown after 72 h of belowground infestation by *Diabrotica virgifera virgifera* (DV), aboveground infestation by *Spodoptera frugiperda* (SF), application of root (MR) or shoot mechanical damage (MS). Non-treated seedlings served as controls (C). The following genes were measured: *BX14* (benzoxazinone synthesis14, Zm00001d004921), *ZRP4-like* (O-methyltransferase, Zm00001d038703), *PR5* (pathogenesis-related protein5, Zm00001d031158), *PR10* (pathogenesis-related protein10), *LOX3* (lipoxygenase, Zm00001d033623), *PPO* (polyphenol oxidase, Zm00001d000001), *BBTI13* (Bowman-Birk type trypsin inhibitor, Zm00001d048660), *CLH* (chlorophyllase1, Zm00001d019758). For qRT-PCR data, fold-change of gene expression level was calculated using the $2^{-\Delta\Delta CT}$ method. The results (threshold cycle values) of the qRT-PCR assays were normalized to the expression level of *ZmCUL* (cullin, Zm00001d024855). For RNA-seq data, gene expression levels were calculated as FPKM (fragments per kilobase of transcript per million fragments mapped).

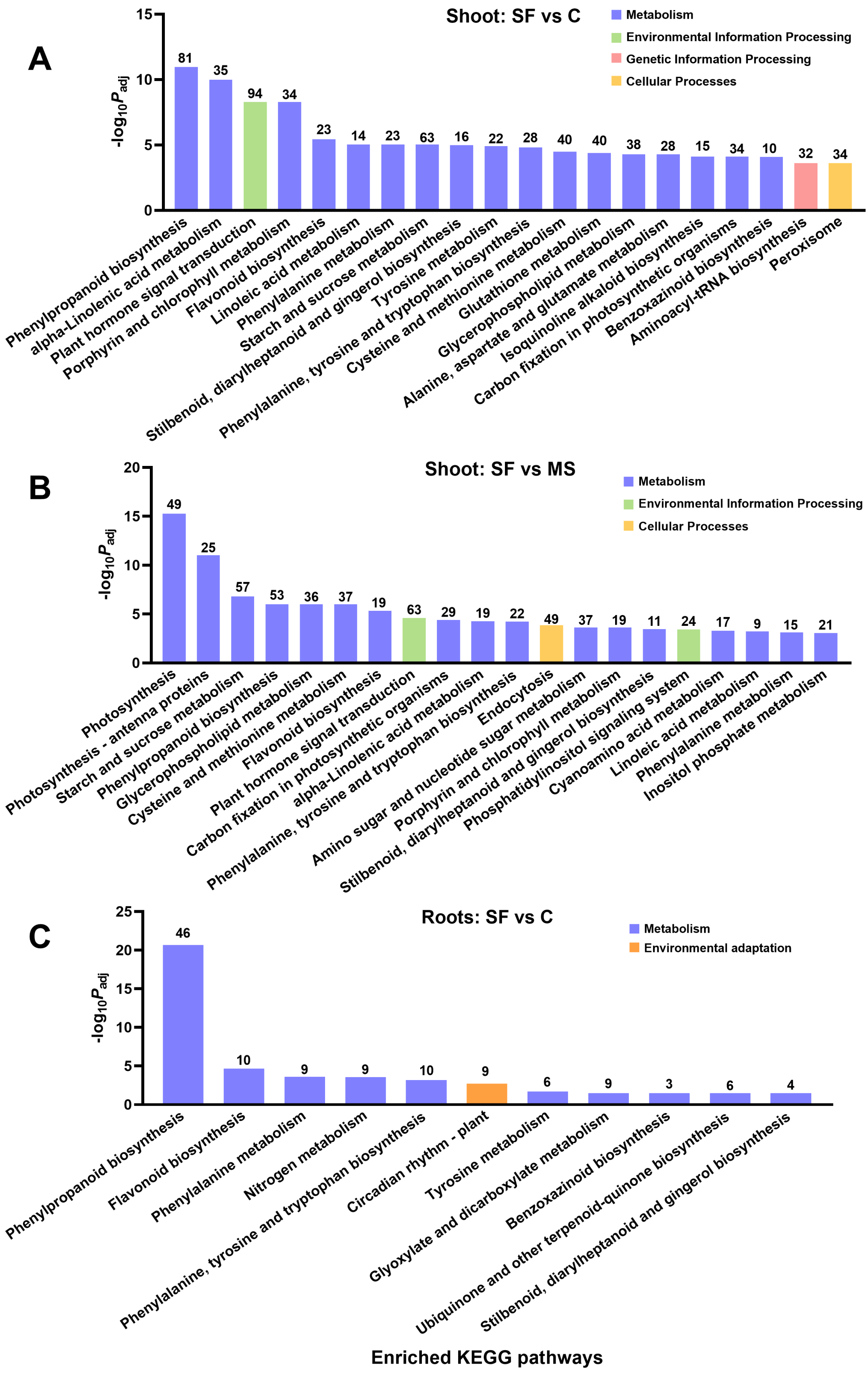

Figure S2 KEGG pathway enrichment analysis of differentially expressed genes (DEGs) in maize induced by *S. frugiperda* herbivory. (**A**) The top 20 enriched KEGG pathways in maize shoot between *S. frugiperda* herbivory (SF) and non-manipulated control (C). (**B**) The top 20 enriched KEGG pathways in maize shoot between *S. frugiperda* herbivory and artificial shoot damage (MS). (**C**) The enriched KEGG pathways in roots of maize seedlings between *S. frugiperda* herbivory and control. Enrichment

scores are shown as -$\log_{10}$(adjusted *P* value). Number of DEGs involved in each pathway are shown above the bar.

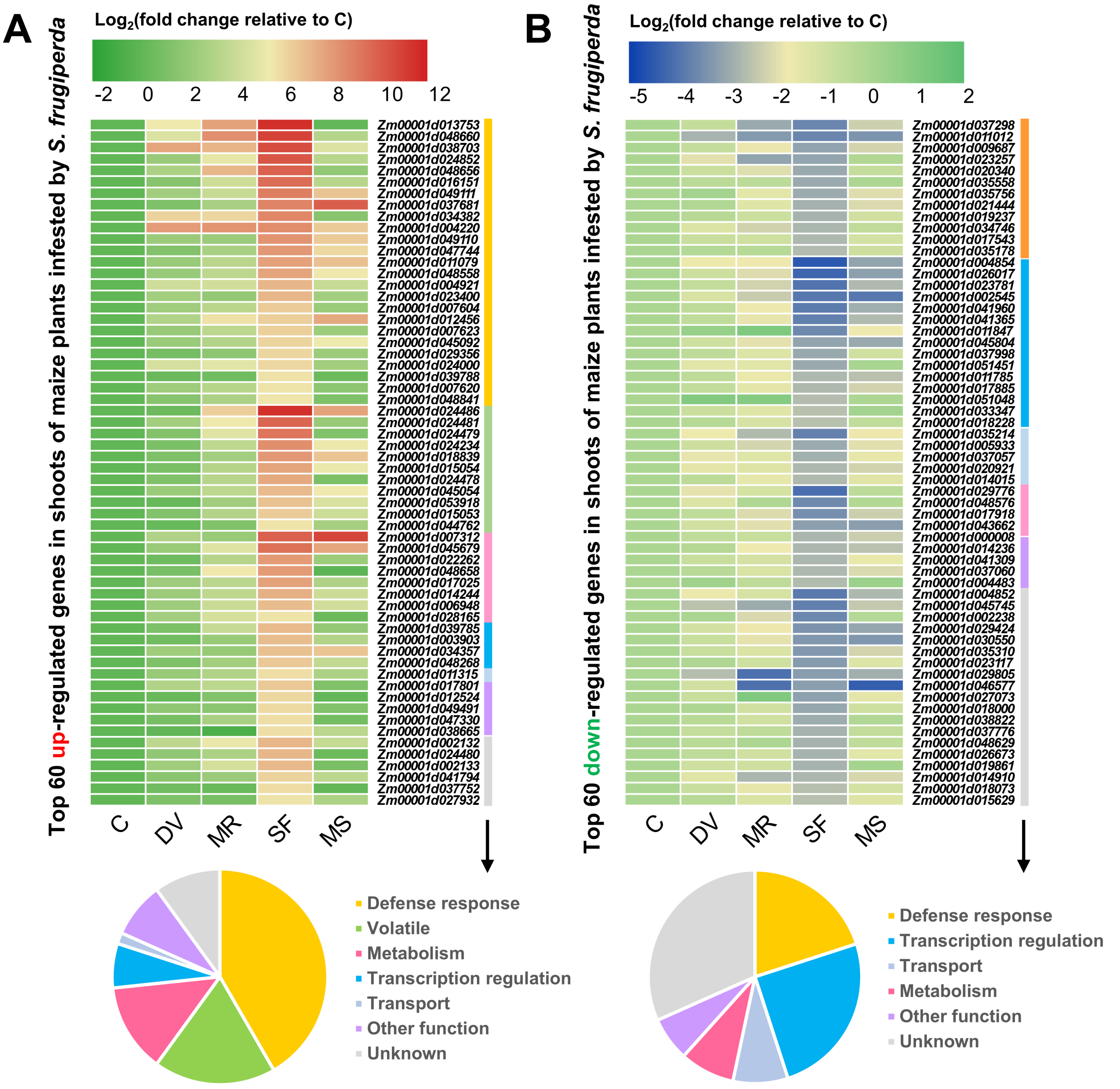

Figure S3 Heatmap of the relative expression levels (fold change after $\log_2$ transformation) of the 60 most up- (**A**) and down-regulated (**B**) genes in maize shoot induced by *Spodoptera frugiperda* feeding. Samples were collected from maize plants that were kept non-manipulated (C, control) or after 72 h of belowground infestation by *Diabrotica virgifera virgifera* (DV), mechanical damage on root (MR), 72 h of aboveground infestation by *Spodoptera frugiperda* (SF), or mechanical damage on shoot (MS). Color coding represents the range of $\log_2$(fold change relative to control). Color bar after gene ID and pie chart under heatmap showing the potential gene function. (**A**) The top 60 most up-regulated genes in shoots induced by *S. frugiperda* feeding, included 25 defense response-related genes (e.g. proteinase inhibitor, β-glucosidase, O-methyltransferase, and genes involved in ethylene-, benzoxazinone-, flavonoid-synthesis) and 11 volatile biosynthesis-related genes (e.g. terpene synthase, germacrene A synthase, dimethylnonatriene synthase, linalool synthase). Several genes, such as Bowman-Birk type trypsin inhibitor (Zm00001d048660), O-methyltransferase *ZRP4* (Zm00001d038703), ethylene biosynthesis-related gene 1-aminocyclopropane-1-carboxylate oxidase 3 (Zm00001d024852), and dirigent protein (Zm00001d004220) were induced by both shoot and root damage, implying their potential role in the systemic defense response to below- and aboveground herbivory. A total of 6 highly

up-regulated genes had no annotation. (**B**) The 60 most down-regulated genes in shoots infested by *S. frugiperda*, included 15 genes involved in transcription regulation, 12 genes involved in defense response, and 5 genes involved in primary metabolism that were highly suppressed by *S. frugiperda* feeding. They comprised a group of MYB-related transcription factors (Zm00001d026017, Zm00001d002545, and Zm00001d037998) and genes involved in the biosynthesis of DIBOA-glucoside (cytochrome P450 71A26, Zm00001d035178), auxin (monooxygenase, Zm00001d021444) and starch (α-amylase 3 chloroplastic, Zm00001d043662). A total of 19 highly down-regulated genes had no annotation.

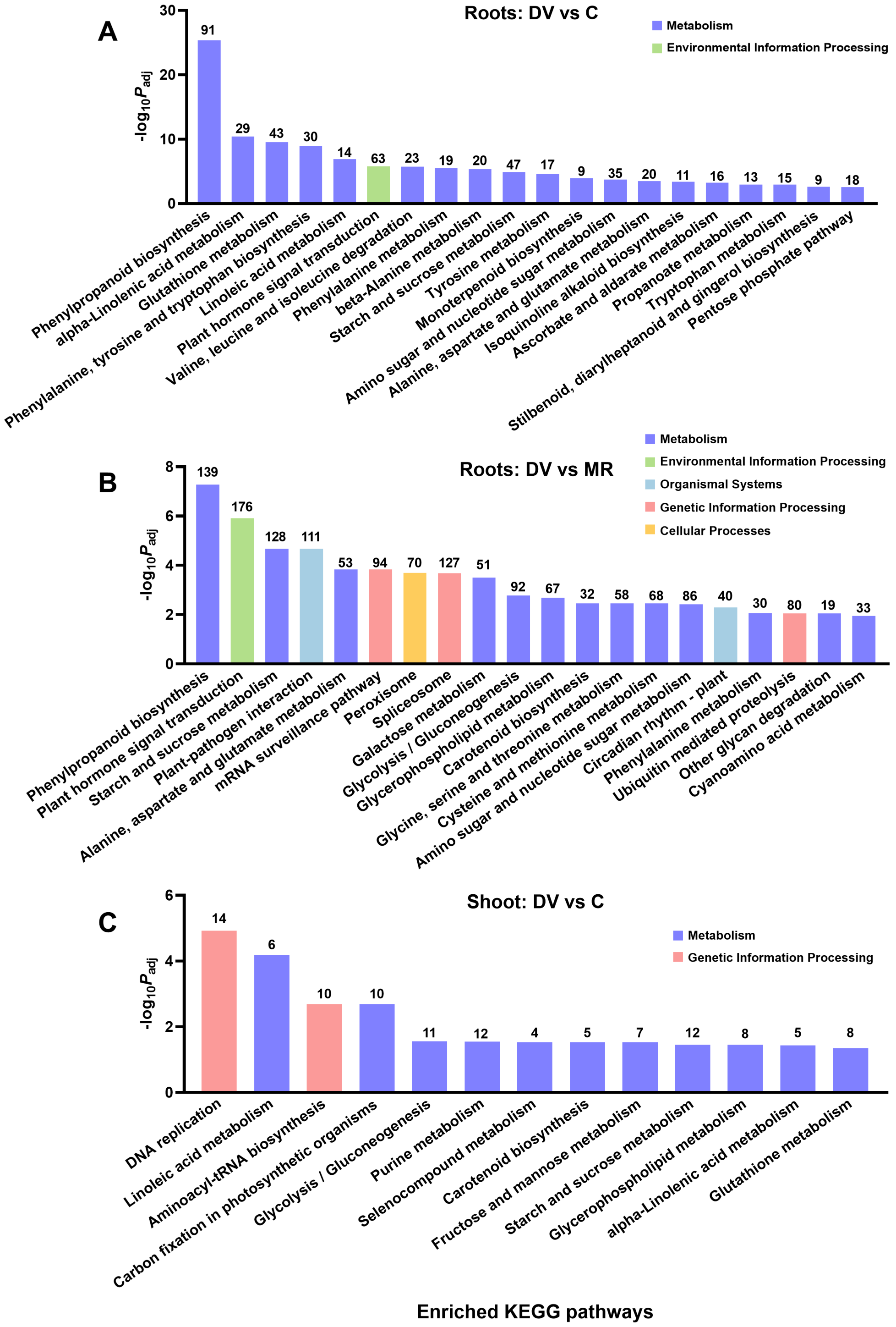

Figure S4 KEGG pathway enrichment analysis of differentially expressed genes (DEGs) in maize induced by *D. v. virgifera* herbivory. (**A**) The top 20 enriched KEGG pathways in maize roots between *D. v. virgifera* herbivory (DV) and non-manipulated control (C). (**B**) The top 20 enriched KEGG pathways

in maize roots between *D. v. virgifera* herbivory and artificial root damage (MR). (**C**) The enriched KEGG pathways in shoot of maize seedling between *D. v. virgifera* herbivory and control. Enrichment scores are shown as -$\log_{10}$(adjusted *P* value). Number of DEGs involved in each pathway are shown above the bar.

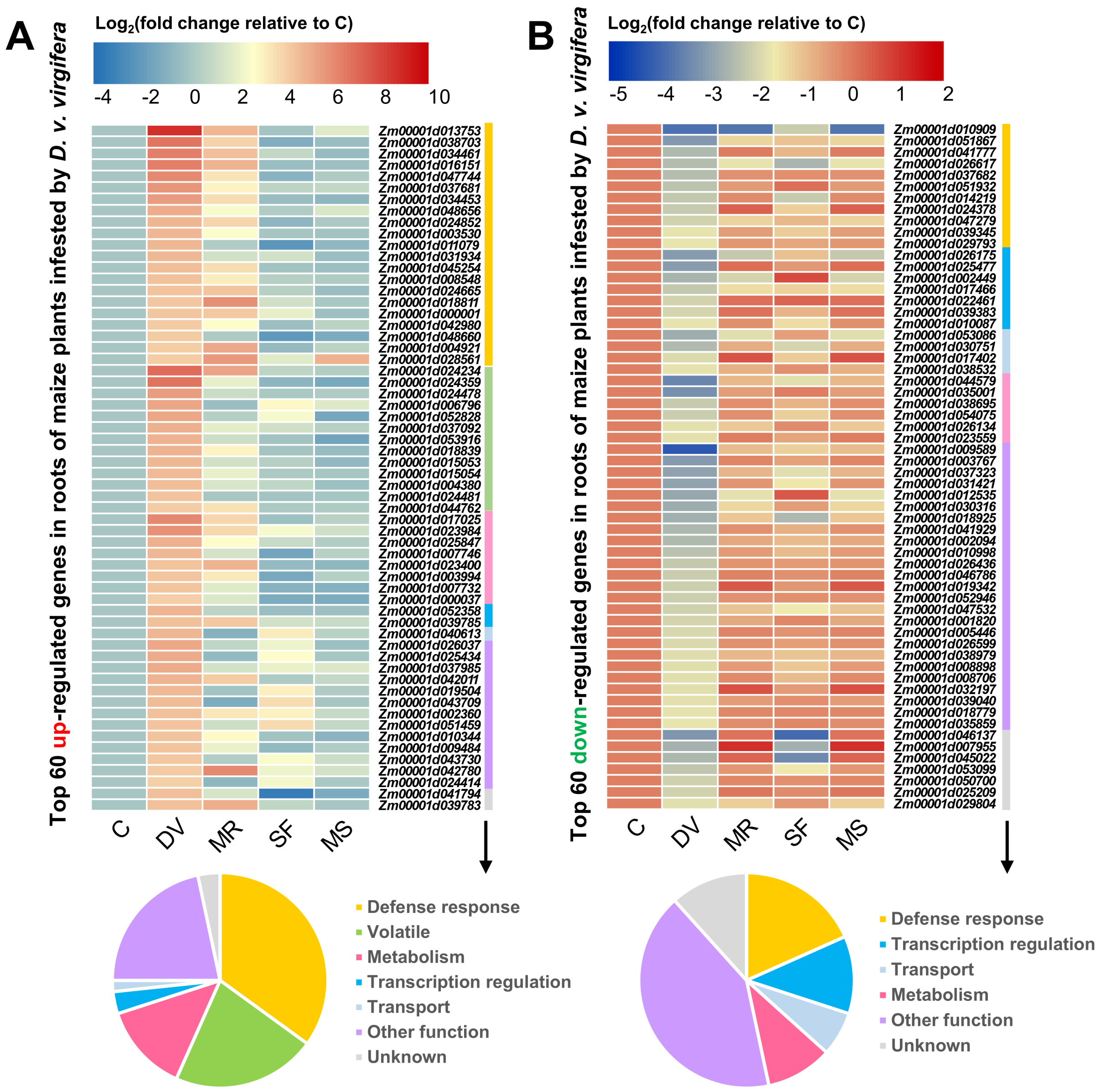

Figure S5 Heatmap of the relative expression levels (fold change after $\log_2$ transformation) of the 60 most up- (**A**) and down-regulated (**B**) genes in maize roots induced by *Diabrotica virgifera virgifera* infestation. Samples were collected from maize plants that were kept non-manipulated (C, control) or after 72 h of belowground infestation by *D. v. virgifera* (DV), mechanical damage on root (MR), 72 h of aboveground infestation by *Spodoptera frugiperda* (SF), or mechanical damage on shoot (MS). Color coding represents the range of $\log_2$(fold change relative to control). Color bar after gene ID and pie chart under heatmap showing the potential gene function. (**A**) The top 60 up-regulated genes in roots induced by *D. v. virgifera* infestation included 21 defense-related genes involved in the biosynthesis of dhurrin (*CYP79A33*, Zm00001d013753), suberin (O-methyltransferase *ZRP4*, Zm00001d038703), DIBOA-glucoside (tryptophan synthase, Zm00001d034461 and Zm00001d034453), isoflavonoid (Zm00001d016151), ethylene (1-aminocyclopropane-1-carboxylate oxidase3, Zm00001d024852), auxin (tyrosine decarboxylase 1, Zm00001d024665), benzoxazinone (benzoxazinone synthesis14,

BX14, Zm00001d004921), proteinase inhibitors (Zm00001d048656, Zm00001d008548, Zm00001d048660) and so on, and 13 volatile emission-related genes (six terpene synthase genes, one β-caryophyllene synthase gene, two salicylate methyltransferase genes, one linalool synthase gene, *CYP92C5*, one NAD(P)-binding Rossmann-fold superfamily protein related to (Z)-3-hexen-1-yl acetate production, and one HXXXD-type acyl-transferase related to volatile benzenoid biosynthesis). Of these direct and indirect defense-related genes in roots, polyphenol oxidase1 (Zm00001d000001), 17.4 kDa class I heat shock protein (Zm00001d028561), NAD(P)-binding Rossmann-fold superfamily protein (Zm00001d006796) and salicylate methyltransferase (Zm00001d052828) were induced by both shoot and root herbivory. Notably, a number of growth and development-related genes such as GDSL esterase/lipase (Zm00001d023984), oil body-associated protein (Zm00001d051459) and several genes coding for embryo protein (Zm00001d025434, Zm00001d037985, Zm00001d043709, Zm00001d002360) and seed maturation protein (Zm00001d026037, Zm00001d024414) were also induced in roots by above- and belowground herbivory. (**B**) From the 60 most down-regulated genes in roots in response to *D. v. virgifera* infestation, many of them were only significantly suppressed by *D. v. virgifera* herbivory, whereas the expression of two potential defense-related genes (Leucine-rich repeat (LRR) family protein, Zm00001d026617; cysteine proteinase inhibitor, Zm00001d014219), one pumilio homolog 3 (Zm00001d018925) and three genes without annotation (Zm00001d046137, Zm00001d007955, Zm00001d045022) were significantly suppressed by both below- and aboveground herbivory. A group of genes involved in photosynthesis (such as chlorophyll a-b binding protein, Zm00001d009589; photosystem I subunit O, Zm00001d003767) and metabolism of prophyrin and chlorophyll (protochlorophyllide reductase1, Zm00001d001820) showed extremely low expression levels in roots compared to their expression in leaves. The expression of these genes in maize roots was down-regulated in response to *D. v. virgifera* infestation.

Table S1 Summary of RNA sequencing and mapping using the maize genome as the reference (in a separate attachment).

Table S2 Primers used for qRT-PCR.

Gene	Locus tag	Forward primer (5'-…-3')	Reverse primer (5'-…-3')
ZmCUL	Zm00001d024855	GAAGAGCCGCAAAGTTATGG	ATGGTAGAAGTGGACGCACC
BX14	Zm00001d004921	AAGACAACTCTATGTGACTGGG	ACTGGTCGGATTTTGTAGTCTT
ZRP4-like	Zm00001d038703	GATAGTCACCAAGGTCACACTC	GAAGAAAGTGCTGAGCATCAAG
PR5	Zm00001d031158	TGCATGCATGGGCTAGTGAT	CGCACACAAATCCAGCTACG
PR10	Zm00001d028816	AGATCACTAAAGCCAAGGAGTC	CATGGTCTAGTTGTAGGCTTCC
LOX3	Zm00001d033623	GCTACGTACGAGCTGGTACATGAA	GCCGCTCTCTTCCCGTTT
PPO	Zm00001d000001	GGTGGACTTAGACTACCTCAAC	TATTTGCTGGGTGTATACGGTG
BBTI13	Zm00001d048660	ACCAAGTAGACGGATGCATATG	TGGTTTATTCAAGGAGAGGCAT
CLH	Zm00001d019758	ACCTCGACATGTTGGACGAC	GACAGGGTCCAGAGTGGTTG

Data S1 to S9 are attached in separate documents

Data S1 Genes detected in all samples. Gene expression levels were shown by Fragments Per Kilobase of transcript per Million mapped reads (FPKMs). Maize shoot (S) and roots (R) were harvested from seedlings that were kept non-manipulated (C, control) or treated with belowground infestation by *D. v. virgifera* (DV), mechanical wounding on root (MR), aboveground infestation by *S. frugiperda* (SF), or mechanical wounding on shoot (MS). NA: no annotation.

Data S2 All DEGs in maize shoot and roots induced by *D. v. virgifera* infestation with a cut-off of two-fold change relative to the control. FC: Fold change. NA: no annotation.

Data S3 All DEGs in maize shoot and roots induced by mechanical root damage with a cut-off of two-fold change relative to the control. FC: Fold change. NA: no annotation.

Data S4 All DEGs in maize shoot and roots induced by *S. frugiperda* infestation with a cut-off of two-fold change relative to the control. FC: Fold change. NA: no annotation.

Data S5 All DEGs in maize shoot and roots induced by mechanical shoot damage with a cut-off of two-fold change relative to the control. FC: Fold change. NA: no annotation.

Data S6 KEGG pathway enrichment analysis of DEGs in the transcriptome of maize induced by different treatments.

Data S7 The top 60 DEGs in maize shoot induced by *S. frugiperda* infestation.

Data S8 The top 60 DEGs in maize roots induced by *D. v. virgifera* infestaion.

Data S9 The gene expression pattern of phytohormones, benzoxazinoids and terpene volatiles.

Supplemental references

Manoli, A., Sturaro, A., Trevisan, S., Quaggiotti, S., and Nonis, A. (2012). Evaluation of candidate reference genes for qPCR in maize. *J. Plant Physiol*. **169**:807–815.

Livak, K. J., and Schmittgen, T. D. (2001). Analysis of relative gene expression data using real-time quantitative PCR and the $2^{-\Delta\Delta CT}$ method. *Methods* **25**:402–408.
